# Crystal structures of three 6-aryl-2-(4-chloro­benz­yl)-5-[(1*H*-indol-3-yl)meth­yl]imidazo[2,1-*b*][1,3,4]thia­diazo­les

**DOI:** 10.1107/S2056989019016050

**Published:** 2020-01-01

**Authors:** Sadashivamurthy Shamanth, Kempegowda Mantelingu, Haruvegowda Kiran Kumar, Hemmige S. Yathirajan, Sabine Foro, Christopher Glidewell

**Affiliations:** a Department of Studies in Chemistry, University of Mysore, Manasagangotri, Mysuru-570 006, India; b Institute of Materials Science, Darmstadt University of Technology, Petersenstrasse 23, D-64287 Darmstadt, Germany; c School of Chemistry, University of St Andrews, St Andrews, Fife KY16 9ST, UK

**Keywords:** synthesis, heterocyclic compounds, imidazo[2,1-*b*][1,3,4]thia­diazo­les, crystal structure, disorder, mol­ecular conformation, hydrogen bonding, supra­molecular assembly

## Abstract

Three title compounds, namely, 2-(4-chloro­benz­yl)-5-[(1*H*-indol-3-yl)meth­yl]-6-phenyl­imidazo[2,1-*b*][1,3,4]thia­diazole, C_26_H_19_ClN_4_S, (I), 2-(4-chloro­benz­yl)-6-(4-fluoro­phen­yl)-5-[(1*H*-indol-3-yl)meth­yl]imidazo[2,1-*b*][1,3,4]thia­diazole, C_26_H_18_ClFN_4_S, (II), and 6-(4-bromo­phen­yl)-2-(4-chloro­benz­yl)-5-[(1*H*-indol-3-yl)meth­yl]imidazo[2,1-*b*][1,3,4]thia­diazole, C_26_H_18_BrClN_4_S, (III), have been prepared using a reductive condensation of indole with the corresponding 6-aryl-2-(4-chloro­benz­yl)imidazo[2,1-*b*][1,3,4]thia­diazole-5-carbaldehydes (aryl = phenyl, 4-fluoro­phenyl or 4-bromo­phen­yl), and their crystal structures have been determined. The asymmetric unit of compound (I) consists of two independent mol­ecules and one of the mol­ecules exhibits disorder of the 4-chloro­benzyl substituent with occupancies 0.6289 (17) and 0.3711 (17). Each type of mol­ecule forms a *C*(8) chain motif built from N—H⋯N hydrogen bonds, which for the fully ordered mol­ecule is reinforced by C—H⋯π inter­actions. In compound (II), the chloro­benzyl unit is again disordered, with occupancies 0.822 (6) and 0.178 (6), and the mol­ecules form *C*(8) chains similar to those in (I), reinforced by C—H⋯π inter­actions involving only the major disorder component. The chloro­benzyl unit in compound (III) is also disordered with occupancies of 0.839 (5) and 0.161 (5). The mol­ecules are linked by a combination of one N—H⋯N hydrogen bond and four C—H⋯π inter­actions, forming a three-dimensional framework.

## Abstract 2

In the crystals of three new 6-aryl-2-(4-chloro­benz­yl)-5-[(1*H*-indol-3-yl)meth­yl]imidazo[2,1-*b*][1,3,4]thia­diazo­les (where aryl is phenyl, 4-fluoro­phenyl or 4-bromo­phen­yl), the mol­ecules are linked by a combination of N—H⋯N and C—H⋯π inter­actions to form chains when the 6-aryl substituent is phenyl or 4-fluoro­phenyl and a three-dimensional framework when the 6-aryl group is 4-bromo­phenyl.

## Chemical context   

**Figure 1:**
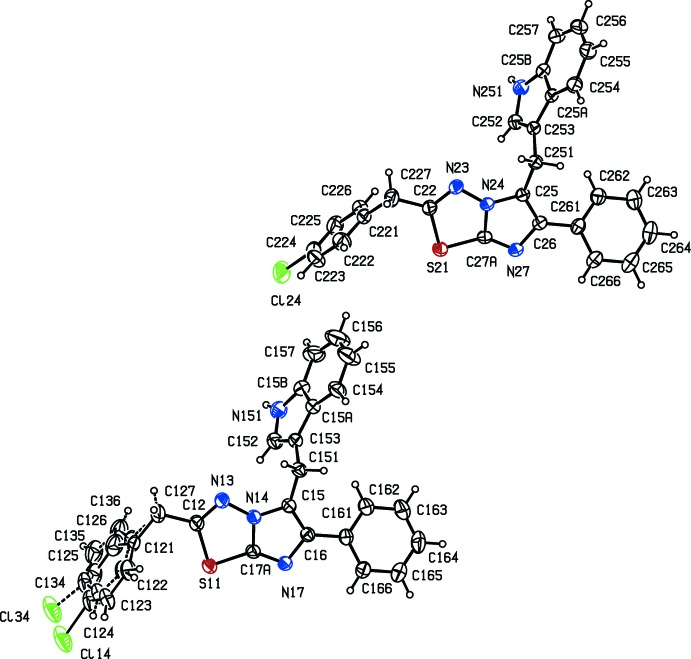
The structures of the two independent mol­ecules of compound (I), showing the atom-labelling scheme and the disorder in one of the mol­ecules. Displacement ellipsoids are drawn at the 30% probability level, and in the disordered fragment, the major disorder component is drawn using full lines and the minor disorder component is drawn using broken lines.

Imidazo[2,1-*b*][1,3,4]thia­diazole is a versatile nucleus for the elaboration of novel heterocyclic compounds as it can readily be substituted at any position of 2, 5 or 6 (Khazi *et al.*, 2011 ▸). A wide range of such derivatives have been evaluated for their biological activities, which encompass anti-cancer, anti-convulsant, anti-fungal, anti-inflammatory and anti-microbial activity, as well as analgesic and anaesthetic properties (Bhongade *et al.*, 2016 ▸). The recently reported indolinone derivative, 6-(4-bromo­phen­yl)-2-(4-chloro­benz­yl)-5-[(1*H*-ind­o­lin-2-one-3-yl)methyl­idene]imidazo[2,1-*b*][1,3,4]thia­diazole (disarib), has been shown to act as a powerful inhibitor of the anti-apoptotic protein BCL2, and to cause significant tumour regression without any significant side effects (Iyer *et al.*, 2016 ▸; Vartak *et al.*, 2016 ▸). With these observations in mind, we have synthesized analogues of disarib, replacing the indolinone substituent with an indolylmethyl unit, while at the same time varying the substituent in the 6-aryl ring, and here we report the preparation, and the mol­ecular and supra­molecular structures of the title three compounds (I)–(III) as shown in Figs. 1 ▸–3 ▸ ▸.
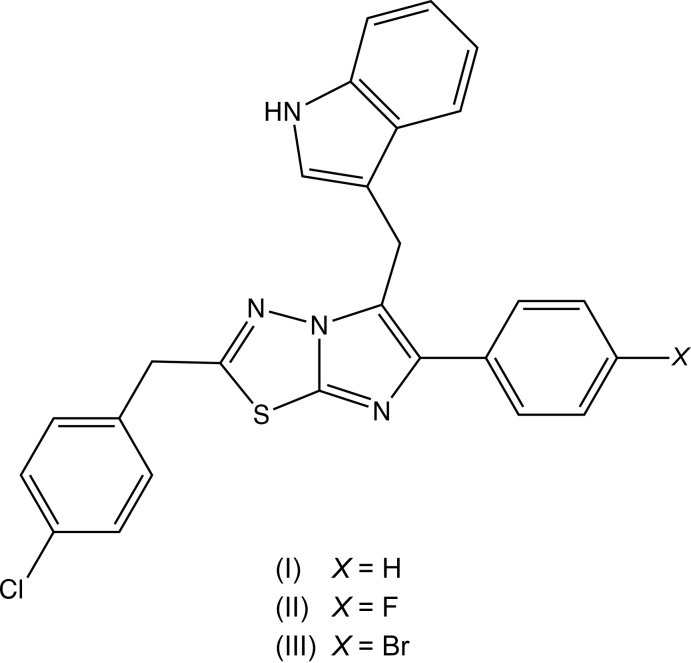


## Structural commentary   

Although compounds (I) and (II) crystallize in the same space group (*P*2_1_/*c*) with *Z*′ = 2 and 1, respectively, compound (III) crystallizes in the non-centrosymmetric space group (*P*2_1_2_1_2_1_). Despite the close similarity in the chemical constitution of compounds (I)–(III), no two of these compounds are isomorphous. None of the mol­ecules exhibits any inter­nal symmetry, so that all of them are conformationally chiral. The centrosymmetric space group for the compounds (I) and (II) show that these have crystallized as conformational racemates. On the other hand, all of the mol­ecules in the crystal of compound (III) in the Sohncke space group have the same conformation; there is no reason to suppose that the crystallization of (III) has involved conformational resolution so that this compound has probably crystallized as a conformational conglomerate (Bernal *et al.*, 1996 ▸). In this conformational enanti­omer, the torsion angle of C5—C6—C61—C62 is −41.3 (6)°, and the reference mol­ecules in (I) and (II) have the same negative sign for this torsion angle (Table 1 ▸).

The asymmetric unit of compound (I) consists of two independent mol­ecules, types 1 and 2, containing atoms S11 and S21, respectively. In the type 1 mol­ecule of compound (I) and in compounds (II) and (III), the 4-chloro­benzyl substituents are each disordered over two sets of atomic sites (Figs. 1 ▸–3 ▸ ▸), having occupancies 0.6289 (17) and 0.3711 (17) for (I), 0.822 (6) and 0.178 (6) for (II), and 0.839 (5) and 0.161 (5) for (III).

The orientation of the chloro­benzyl unit relative to that of the central imidazo[2,1-*b*][1,3,4]thia­diazole ring system differs quite significantly between compounds (I) and (II) on the one hand and with that in compound (III) on the other, as indicated by the torsion angles S*x*1—C*x*2—C*x*27—C*x*21/C*x*31 (Table 1 ▸). This may be associated with the observation that this unit in (I) and (II) acts as a hydrogen-bond donor but not as an acceptor, while in (III) as an acceptor but not a donor (Table 2 ▸). Similarly, the orientation of the indole­methyl­ene group relative to the imidazo[2,1-*b*][1,3,4]thia­diazole unit shows considerable differences between compounds (I) and (II) on the one hand and compound (III) on the other, as shown by the torsion angles N*x*4—C*x*5—C*x*51—C*x*53 and C*x*5—C*x*51—C*x*53—C*x*52 (Table 1 ▸), although the indole unit acts as both a donor and an acceptor of hydrogen bonds in all three compounds (Table 2 ▸). A small change in a single mono-atomic substituent thus effects significant changes in both the crystallization characteristics and the mol­ecular conformations in compounds (I)–(III).

## Supra­molecular features   

**Figure 4:**
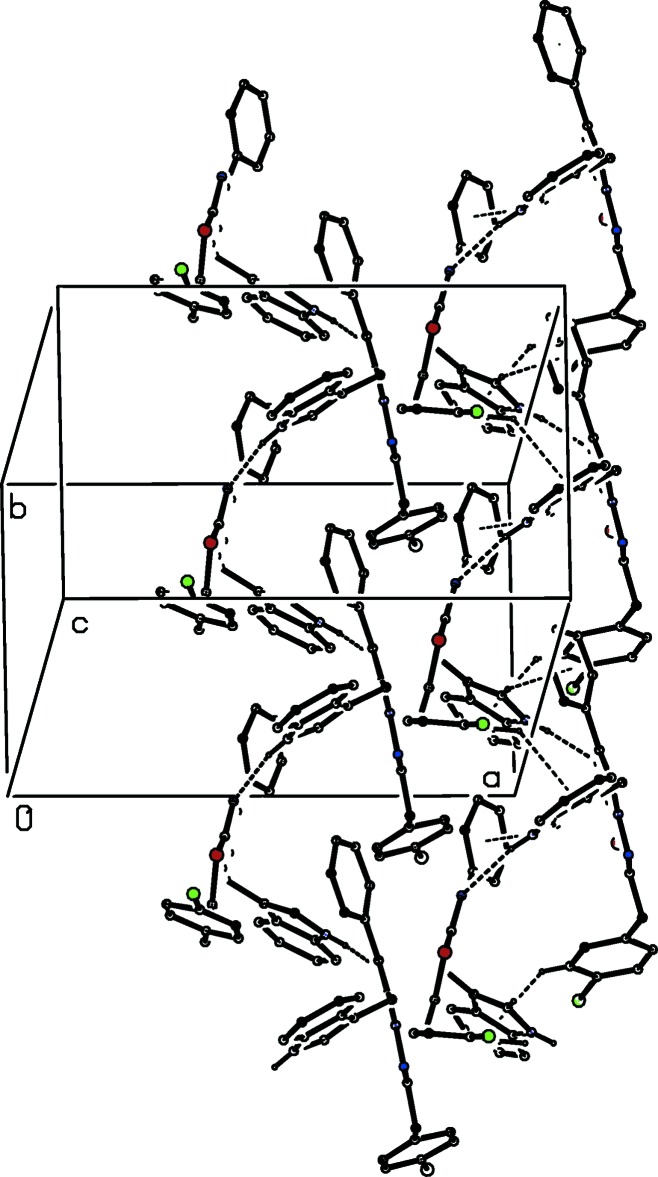
Part of the crystal structure of compound (I), showing two *C*(8) chains running along the [010] direction, one built from N—H⋯N hydrogen bonds and the other from N—H⋯N and C—H⋯π inter­actions shown as dashed lines. For the sake of clarity, the minor disorder component and the H atoms not involved in the inter­actions have been omitted.

In the crystal of compound (I), the mol­ecules of type 1, which are related by a 2_1_ screw axis, are linked by N—H⋯N hydrogen bonds, forming a *C*(8) chain motif running along [010] (Fig. 4 ▸). Similarly, the type 2 mol­ecules, which are related by another 2_1_ screw axis, form a second *C*(8) chain along [010]. These chains differ in that the second chain is reinforced by two C—H⋯π inter­actions, whereas in the first chain, only the minor disorder component takes part in such an inter­action; in the major disorder component, the shortest inter­molecular H⋯*Cg* distance exceeds 3.3 Å [H126⋯*Cg*1^i^ = 3.33 Å; *Cg*1 is the centroid of the ring C15*A*/C154–C157/C15*B*; symmetry code: (i) 1 − *x*, −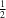 + *y*, 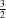 − *z*] .

**Figure 5:**
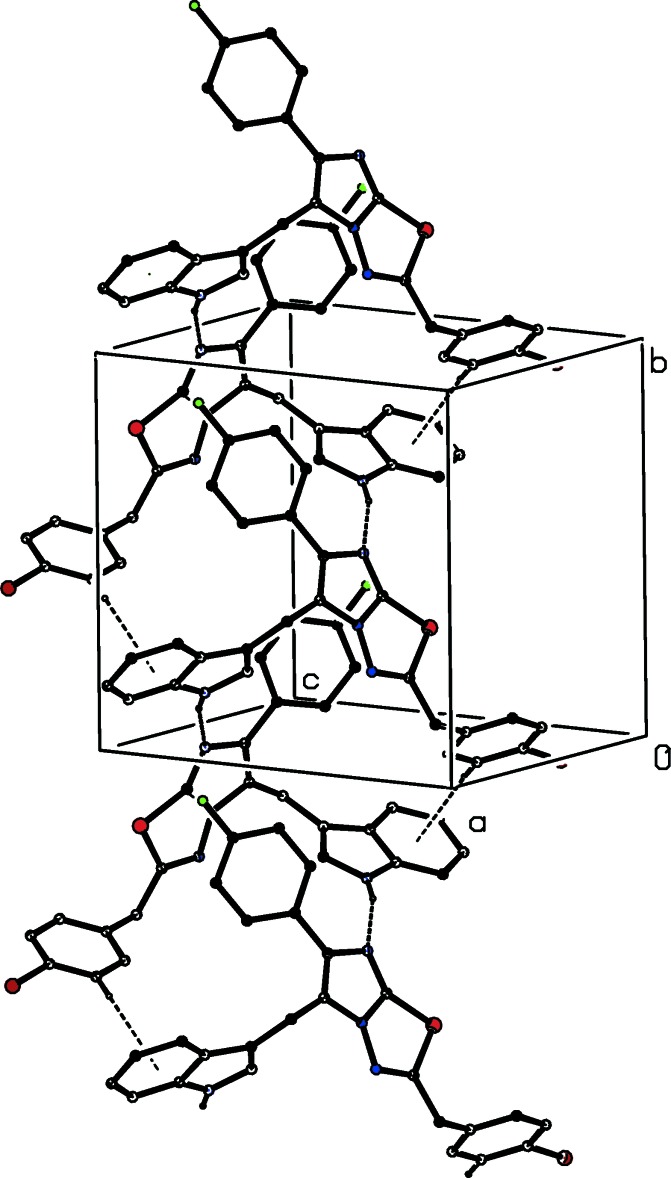
Part of the crystal structure of compound (II), showing a mol­ecular chain running along the [010] direction formed *via* N—H⋯N and C—H⋯π inter­actions (dashed lines). The minor disorder component and the H atoms not involved in the inter­actions have been omitted.

In the crystal of compound (II), there are an N—H⋯N hydrogen bond and a C—H⋯π inter­action (Table 2 ▸); the C—H⋯π inter­action is present only for the major disorder component. The N—H⋯N hydrogen bond links the mol­ecules, which are related by a 2_1_ screw axis, into a *C*(8) chain running along [010]. This hydrogen bond is augmented by the C—H⋯π inter­action (Fig. 5 ▸). There are no direction-specific inter­actions between adjacent chains, so that the supra­molecular aggregation is one-dimensional.

**Figure 6:**
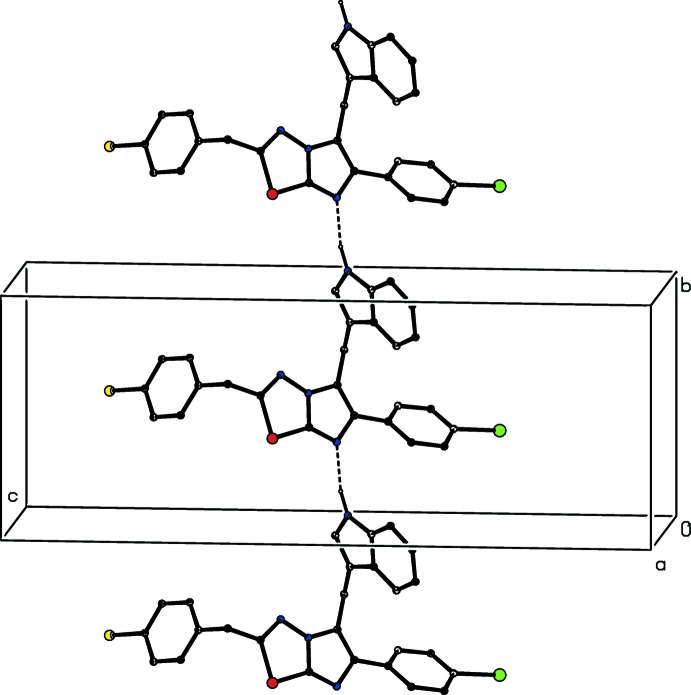
Part of the crystal structure of compound (III), showing a *C*(8) chain running along the [010] direction built from N—H⋯N hydrogen bonds (dashed lines). The minor disorder component and the H atoms bonded to C atoms have been omitted.

**Figure 7:**
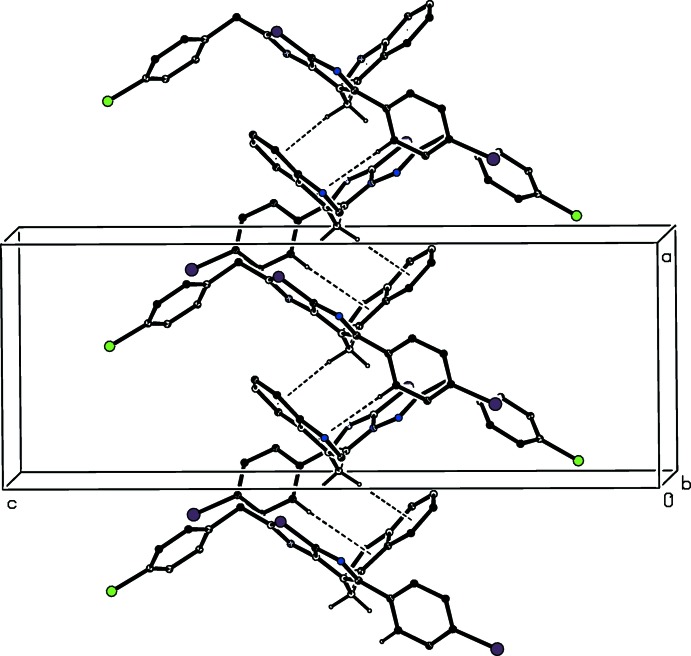
Part of the crystal structure of compound (III), showing a chain running along the [100] direction built from C—H⋯π inter­actions (dashed lines). The minor disorder component and the H atoms not involved in the motif have been omitted.

**Figure 8:**
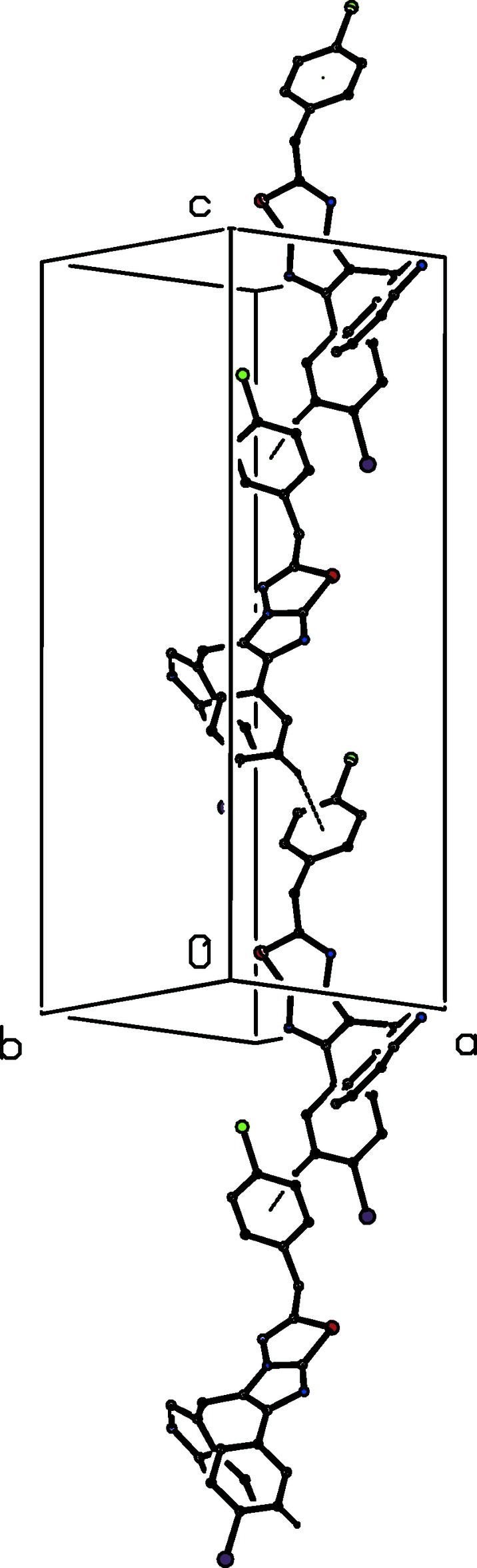
Part of the crystal structure of compound (III), showing a chain running along the [001] direction built from C—H⋯π inter­actions (dashed lines). The minor disorder component and the H atoms not involved in the motif have been omitted.

The supra­molecular structure of compound (III) contains an N—H⋯N hydrogen bond, as in (I) and (II), along with four C—H⋯π inter­actions, which have rather long H⋯*Cg* distances (Table 2 ▸). The N—H⋯N hydrogen bond links mol­ecules, which are related by translation, to form a *C*(8) chain along [010] (Fig. 6 ▸). Two C—H⋯π inter­actions, involving atoms C51 and C62 (Table 2 ▸), cooperatively link mol­ecules, which are related by a 2_1_ screw axis along the *x* axis, to form a chain along the [100] direction (Fig. 7 ▸). Finally, two C—H⋯π inter­actions involving atoms C65 and C62 form similar contacts to the aryl rings of both disorder components, generating a chain of mol­ecules related by a 2_1_ screw axis running along [001] (Fig. 8 ▸). The combination of chains running along the [100], [010] and [001] directions suffices to link all of the mol­ecules into a three-dimensional framework structure. As with the crystallization characteristics and the mol­ecular conformations, simple changes of substituent between (I), (II) and (III) effect marked changes in the supra­molecular aggregation.

## Database survey   

**Figure 9:**
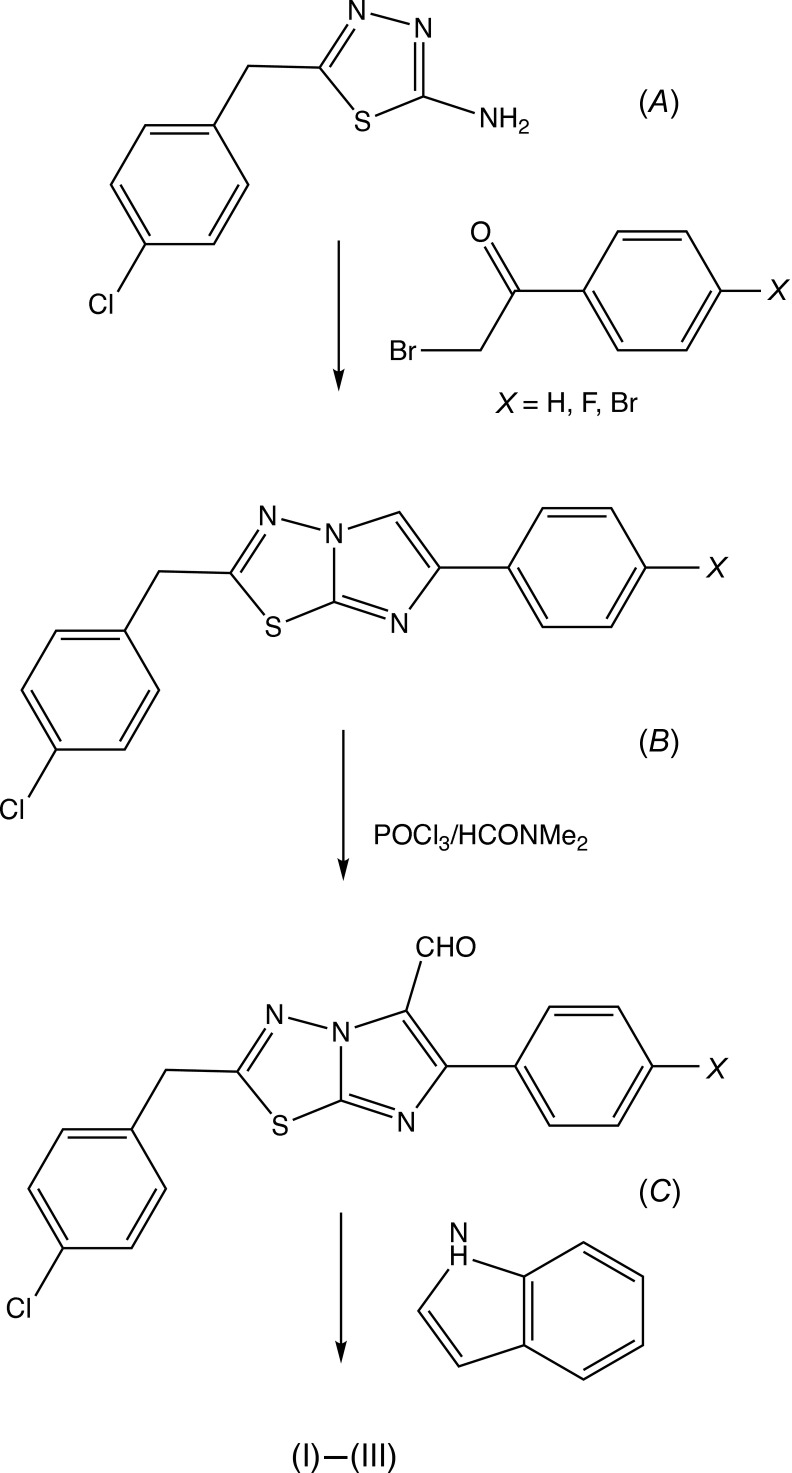
The reaction sequence used for the synthesis of compounds (I)–(III).

The structures of a number of analogues of the inter­mediates, (*B*) in Fig. 9 ▸, have been reported. These include 2-(4-fluoro­benz­yl)-6-(4-nitro­phen­yl)imidazo[2,1-*b*][1,3,4]thia­diazole (Banu *et al.*, 2010*b* ▸), 2-6-(4-bromo­phen­yl)-(4-fluoro­benz­yl)imidazo[2,1-*b*][1,3,4]thia­diazole (Banu, Begum *et al.*, 2011 ▸), 2-(4-fluoro­benz­yl)-6-(4-meth­oxy­phen­yl)imidazo[2,1-*b*][1,3,4]thia­diazole (Banu *et al.*, 2013 ▸), 2-(4-fluoro­benz­yl)-6-phenyl­imidazo[2,1-*b*][1,3,4]thia­diazole (Banu *et al.*, 2014 ▸), 6-(4-chloro­phen­yl)-(4-fluoro­benz­yl)imidazo[2,1-*b*][1,3,4]thia­diazole (Banu *et al.*, 2014 ▸), which is isostructural with the 6-(4-bromo­phen­yl) analogue (Banu *et al.*, 2011 ▸) and 2-benzyl-6-(4-chloro­phen­yl)imidazo[2,1-*b*][1,3,4]thia­diazole (Anil Kumar & Kokila, 2016 ▸). The structures of two 5-carbaldehyde derivatives have also been reported, which are analogues of the inter­mediates, (*C*) in Fig. 9 ▸, namely, 2-cyclo­hexyl-6-(4-bro­mo­phenyl)imidazo[2,1-*b*][1,3,4]thia­diazole-5-carbaldehyde (Shahina Begum *et al.*, 2008 ▸) and 2-(4-fluoro­benz­yl)-6-phenyl­imidazo[2,1-*b*][1,3,4]thia­diazole-5-cabaldehyde (Banu *et al.*, 2010*a* ▸). The reported structures for analogues of the products (I)–(III) carrying heterocyclic substituents at position 5 are few, but they include 5-(morpholin-4-ylmeth­yl)-2-(phen­oxy­meth­yl)-6-phenyl­imidazo[2,1-*b*][1,3,4]thia­diazole (Da *et al.*, 2012 ▸) and 2-(4-fluoro­benz­yl)-6-(4-meth­oxy­phen­yl)-5-(morpholin-4-ylmeth­yl)imidazo[2,1-*b*][1,3,4]thia­diazole (Banu *et al.*, 2013 ▸). Finally, we note an isostructural pair of compounds carrying 1,2-benzoxazole substituents at position 2 of the imidazo[2,1-*b*][1,3,4]thia­diazole unit, namely, 3-{[6-(4-chloro­phen­yl)imid­azo[2,1-*b*][1,3,4]thia­diazol-2-yl]meth­yl}-1,2-benzoxazole (Banu, Ziaulla *et al.*, 2011*b* ▸) and its 6-(4-bromo­phen­yl) analogue (Banu, Ziaulla *et al.*, 2011*a* ▸).

## Synthesis and crystallization   

The title compounds, C_26_H_18_Cl*X*N_4_S (*X* = H, F, Br), were prepared in a three-step sequence, as shown in Fig. 9 ▸, from the readily accessible precursor 2-amino-5-(4-chloro­benz­yl)-[1,3,4]thia­diazole, (*A*), using an established methodology (Appleton *et al.*, 1993 ▸; Karki *et al.*, 2011 ▸; Iyer *et al.*, 2016 ▸) by means of successive condensation with a substituted phenacyl bromide to form the 2,5-disubstituted imidazo[2,1-*b*][1,3,4]thia­diazo­les, (*B*), followed by Vilsmeier–Haack formyl­ation to give the corresponding 5-carbaldehydes, (*C*), and finally reductive condensation with indole in the presence of tri­ethyl­silane and tri­fluoro­acetic acid (Appleton *et al.*, 1993 ▸) to form the products (I)–(III). We have also prepared the 4-chloro­phenyl analogue (*X* = Cl), but unfortunately no crystals of this compound have yet been obtained, only a viscous gum.

Compound (I), *X* = H: yield 58%, m.p. 493–495 K; HRMS found 455.0000. C_26_H_19_^35^ClN_4_S requires for (*M* + H)^+^ 455.1019. Compound (II), *X* = F: yield 48%, m.p. 483–485 K; HRMS found 473.0620, C_26_H_18_^35^ClFN_4_S requires for (*M* + H)^+^ 473. 0925. Compound (III), *X* = Br: yield 52%, m.p. 393–395 K; HRMS found 532.8687, C_26_H_18_^79^Br^35^ClN_4_S requires for (*M* + H)^+^ 533.0124. Crystals of (I)–(III) suitable for single-crystal X-ray diffraction were grown by slow evaporation in the presence of air of solutions in ethyl acetate at ambient temperature. 4-Chloro­phenyl analogue (*X* = Cl): yield 48%, m.p. 503–505 K; HRMS found 488.914, C_26_H_18_^35^Cl_2_N_4_S requires for (*M* + H)^+^ 489.0629.

## Refinement   

Crystal data, data collection and structure refinement details are summarized in Table 3 ▸. In each compound, the chloro­benzyl unit was disordered over two sets of atomic sites having unequal occupancies. In each case, the bond lengths and the 1,3-distances in the minor disorder component were restrained to be the same as the equivalent distances in the major disorder component, subject to s.u. values of 0.01 and 0.02 Å, respectively, and the anisotropic displacement parameters for pairs of partial-occupancy atoms occupying essentially the same physical space were constrained to be equal. In addition, it was found necessary to constrain the minor component of the disordered chloro­benzyl group in (II) to be planar. Apart from those in the minor disorder components, all H atoms were located in difference maps. The H atoms bonded to C atoms were then treated as riding atoms in geometrically idealized positions with C—H distances 0.93 Å (aromatic and heteroaromatic) or 0.97 Å (CH_2_), and with *U*_iso_(H) = 1.2*U*_eq_(C). For the H atoms bonded to N atoms, the atomic coordinates were refined with *U*_iso_(H) = 1.2*U*_eq_(N), giving refined N—H distances of 0.83 (3)–0.99 (5) Å. On this basis, the refined occupancies of the disorder components were 0.6289 (17) and 0.3711 (17) for (I), 0.822 (6) and 0.178 (6) for (II), and 0.839 (5) and 0.161 (5) for (III).

## Supplementary Material

Crystal structure: contains datablock(s) global, I, II, III. DOI: 10.1107/S2056989019016050/is5527sup1.cif

Structure factors: contains datablock(s) I. DOI: 10.1107/S2056989019016050/is5527Isup2.hkl

Structure factors: contains datablock(s) II. DOI: 10.1107/S2056989019016050/is5527IIsup3.hkl

Structure factors: contains datablock(s) III. DOI: 10.1107/S2056989019016050/is5527IIIsup5.hkl

Supporting information file. DOI: 10.1107/S2056989019016050/is5527Isup5.cml

Supporting information file. DOI: 10.1107/S2056989019016050/is5527IIsup6.cml

Supporting information file. DOI: 10.1107/S2056989019016050/is5527IIIsup7.cml

CCDC references: 1968781, 1968780, 1968779

Additional supporting information:  crystallographic information; 3D view; checkCIF report

## Figures and Tables

**Figure 2:**
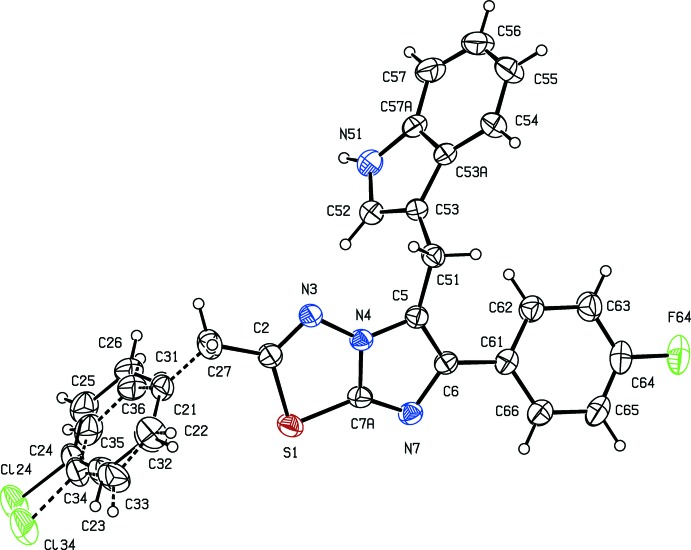
The mol­ecular structure of compound (II), showing the atom-labelling scheme and the disorder. Displacement ellipsoids are drawn at the 30% probability level, and in the disordered fragment, the major disorder component is drawn using full lines and the minor disorder component is drawn using broken lines.

**Figure 3:**
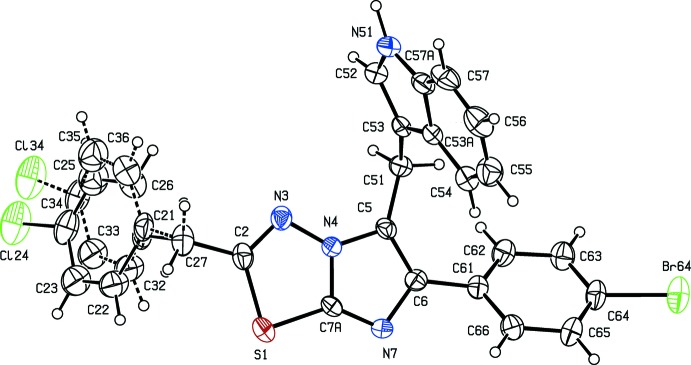
The mol­ecular structure of compound (III), showing the atom-labelling scheme and the disorder. Displacement ellipsoids are drawn at the 30% probability level, and in the disordered fragment, the major disorder component is drawn using full lines and the minor disorder component is drawn using broken lines.

**Table 1:** Selected torsion angles (°) for compounds (I)–(III)

Parameter	(I) type 1	(I) type 2	(II)	(III)
	*x* = 1	*x* = 2	*x* = nul	*x* = nul
S*x*1—C*x*2—C*x*27—C*x*21	−4.1 (6)	−26.9 (4)	−23.4 (5)	−89.6 (7)
S*x*1—C*x*2—C*x*37—C*x*31	−9.7 (11)		−19.8 (17)	−98 (2)
C*x*2—C*x*27—C*x*21—C*x*22	82.4 (18)	111.2 (3)	91.7 (4)	87 (2)
C*x*2—C*x*37—C*x*31—C*x*32	71 (3)		96.2 (17)	63 (10)
N*x*4—C*x*5—C*x*51—C*x*53	−83.8 (3)	−84.2 (3)	−86.4 (3)	66.0 (5)
C*x*5—C*x*51—C*x*53—C*x*52	21.5 (4)	14.9 (3)	27.4 (4)	−133.1 (4)
C*x*5—C*x*6—C*x*61—C*x*62	−24.7 (4)	−33.7 (3)	−27.0 (4)	−41.3 (6)

**Table 2:** Hydrogen bond geometries (Å, °) for compounds (I)–(III) *Cg*1–*Cg*7 represent the centroids of the C15*A*/C154–C157/C15*B*, C25*A*/C254–C257/C25*B*, C261–C266, C53*A*/C54–C57/C57*A*, N51/C52/C53/C53*A*C54/C57*A*, C21–C26 and C31–C36 rings, respectively.

Compound	*D*—H⋯*A*	*D*—H	H⋯*A*	*D*⋯*A*	*D*—H⋯*A*
(I)	N151—H151⋯N17^i^	0.83 (3)	2.11 (3)	2.912 (3)	162 (3)
	N251—H251⋯N27^ii^	0.83 (3)	2.27 (3)	3.087 (3)	167 (3)
	C135—H135⋯*Cg*1^i^	0.93	2.52	3.272 (11)	138
	C225—H225⋯*Cg*2^ii^	0.93	2.87	3.568 (4)	133
	C252—H252⋯*Cg*3^ii^	0.93	2.77	3.568 (3)	134
(II)	N51—H51⋯N7^i^	0.86 (3)	2.27 (3)	3.102 (3)	165 (3)
	C25—H25⋯*Cg*4^i^	0.93	2.75	3.637 (5)	161
(III)	N51—H51⋯N7^iii^	0.99 (5)	1.97 (5)	2.941 (5)	166 (4)
	C51—H51*A*⋯*Cg*4^iv^	0.97	2.97	3.699 (5)	133
	C62—H62⋯*Cg*5^iv^	0.93	2.91	3.757 (5)	152
	C65—H65⋯*Cg*6^v^	0.93	2.82	3.412 (7)	123
	C62—H62⋯*Cg*7^v^	0.93	2.91	3.60 (3)	131

Symmetry codes: (i) 1 − *x*, −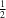 + *y*, 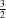 − *z*; (ii) 2 − *x*, −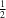 + *y*, 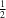 − *z*; (iii) *x*, 1 + *y*, *z*; (iv) −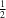 + *x*, 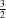 − *y*, 1 − *z*; (v) 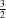 − *x*, 1 − *y*, −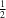 + *z*.

**Table 3:** Experimental details

	(I)	(II)	(III)
Crystal data			
Chemical formula	C_26_H_19_ClN_4_S	C_26_H_18_ClFN_4_S	C_26_H_18_BrClN_4_S
*M*_r_	454.96	472.95	533.85
Crystal system, space group	Monoclinic, *P*2_1_/*c*	Monoclinic, *P*2_1_/*c*	Orthorhombic, *P*2_1_2_1_2_1_
Temperature (K)	302	296	296
*a*, *b*, *c* (Å)	16.456 (7), 10.420 (2), 26.391 (7)	15.340 (1), 11.1619 (7), 15.385 (1)	9.5735 (8), 9.6860 (9), 25.644 (2)
α, β, γ (°)	90, 90.031 (12), 90	90, 119.48 (1), 90	90, 90, 90
*V* (Å^3^)	4525 (2)	2293.2 (3)	2377.9 (4)
*Z*	8	4	4
Radiation type	Mo *K*α	Mo *K*α	Mo *K*α
μ (mm^−1^)	0.28	0.29	1.95
Crystal size (mm)	0.33 × 0.31 × 0.28	0.46 × 0.44 × 0.20	0.48 × 0.44 × 0.44

Data collection			
Diffractometer	Bruker *SMART* X2S benchtop	Oxford Diffraction Xcalibur with Sapphire CCD	Oxford Diffraction Xcalibur with Sapphire CCD
Absorption correction	Multi-scan (*SADABS*; Bruker, 2017 ▸)	Multi-scan (*CrysAlis RED*; Oxford Diffraction, 2009 ▸)	Multi-scan (*CrysAlis RED*; Oxford Diffraction, 2009 ▸)
*T*_min_, *T*_max_	0.845, 0.924	0.768, 0.944	0.368, 0.424
No. of measured, independent and observed [*I* > 2σ(*I*)] reflections	55921, 10420, 7091	16612, 5041, 3161	10501, 4645, 3140
*R*_int_	0.036	0.025	0.030
(sin θ/λ)_max_ (Å^−1^)	0.651	0.651	0.658

Refinement			
*R*[*F* ^2^ > 2σ(*F* ^2^)], *wR*(*F* ^2^), *S*	0.060, 0.177, 1.02	0.050, 0.131, 1.01	0.045, 0.100, 1.02
No. of reflections	10420	5041	4645
No. of parameters	605	323	323
No. of restraints	18	23	18
H-atom treatment	H atoms treated by a mixture of independent and constrained refinement	H atoms treated by a mixture of independent and constrained refinement	H atoms treated by a mixture of independent and constrained refinement
Δρ_max_, Δρ_min_ (e Å^−3^)	0.77, −0.60	0.26, −0.24	0.40, −0.51
Absolute structure	–	–	Flack *x* determined using 943 quotients [(*I* ^+^)−(*I* ^−^)]/[(*I* ^+^)+(*I* ^−^)] (Parsons *et al.*, 2013 ▸)
Absolute structure parameter	–	–	0.014 (5)

Computer programs: *APEX2* (Bruker, 2012 ▸), *SAINT* (Bruker, 2017 ▸), *CrysAlis CCD* and *CrysAlis RED* (Oxford Diffraction, 2009 ▸), *SHELXT* (Sheldrick, 2015*a* ▸), *SHELXL2014* (Sheldrick, 2015*b* ▸) and *PLATON* (Spek, 2009 ▸).

## supplementary crystallographic information

### 2-(4-Chlorobenzyl)-5-[(1H-indol-3-yl)methyl]-6-phenylimidazo[2,1-b][1,3,4]thiadiazole (I) Crystal data

**Table:**

C_26_H_19_ClN_4_S	*F*(000) = 1888
*M**_r_* = 454.96	*D*_x_ = 1.336 Mg m^−^^3^
Monoclinic, *P*2_1_/*c*	Mo *K*α radiation, λ = 0.71073 Å
*a* = 16.456 (7) Å	Cell parameters from 12458 reflections
*b* = 10.420 (2) Å	θ = 2.1–29.5°
*c* = 26.391 (7) Å	µ = 0.28 mm^−^^1^
β = 90.031 (12)°	*T* = 302 K
*V* = 4525 (2) Å^3^	Block, colourless
*Z* = 8	0.33 × 0.31 × 0.28 mm

### 2-(4-Chlorobenzyl)-5-[(1H-indol-3-yl)methyl]-6-phenylimidazo[2,1-b][1,3,4]thiadiazole (I) Data collection

**Table:**

Bruker SMART X2S benchtop diffractometer	10420 independent reflections
Radiation source: fine-focus sealed tube	7091 reflections with *I* > 2σ(*I*)
Graphite monochromator	*R*_int_ = 0.036
Detector resolution: 7.3910 pixels mm^-1^	θ_max_ = 27.6°, θ_min_ = 2.1°
φ and ω scans	*h* = −21→21
Absorption correction: multi-scan (SADABS; Bruker, 2017)	*k* = −12→13
*T*_min_ = 0.845, *T*_max_ = 0.924	*l* = −34→34
55921 measured reflections	

### 2-(4-Chlorobenzyl)-5-[(1H-indol-3-yl)methyl]-6-phenylimidazo[2,1-b][1,3,4]thiadiazole (I) Refinement

**Table:**

Refinement on *F*^2^	Primary atom site location: difference Fourier map
Least-squares matrix: full	Hydrogen site location: mixed
*R*[*F*^2^ > 2σ(*F*^2^)] = 0.060	H atoms treated by a mixture of independent and constrained refinement
*wR*(*F*^2^) = 0.177	*w* = 1/[σ^2^(*F*_o_^2^) + (0.0741*P*)^2^ + 2.5289*P*] where *P* = (*F*_o_^2^ + 2*F*_c_^2^)/3
*S* = 1.02	(Δ/σ)_max_ = 0.001
10420 reflections	Δρ_max_ = 0.77 e Å^−^^3^
605 parameters	Δρ_min_ = −0.59 e Å^−^^3^
18 restraints	

### 2-(4-Chlorobenzyl)-5-[(1H-indol-3-yl)methyl]-6-phenylimidazo[2,1-b][1,3,4]thiadiazole (I) Special details

**Table:**

Geometry. All esds (except the esd in the dihedral angle between two l.s. planes) are estimated using the full covariance matrix. The cell esds are taken into account individually in the estimation of esds in distances, angles and torsion angles; correlations between esds in cell parameters are only used when they are defined by crystal symmetry. An approximate (isotropic) treatment of cell esds is used for estimating esds involving l.s. planes.

### 2-(4-Chlorobenzyl)-5-[(1H-indol-3-yl)methyl]-6-phenylimidazo[2,1-b][1,3,4]thiadiazole (I) Fractional atomic coordinates and isotropic or equivalent isotropic displacement parameters (Å^2^)

**Table:**

	*x*	*y*	*z*	*U*_iso_*/*U*_eq_	Occ. (<1)
S11	0.31032 (5)	0.27517 (7)	0.84740 (2)	0.0673 (2)	
C12	0.30411 (19)	0.1424 (2)	0.80654 (10)	0.0657 (7)	
N13	0.31605 (14)	0.16395 (19)	0.75885 (8)	0.0592 (5)	
N14	0.33131 (12)	0.29274 (18)	0.75295 (7)	0.0500 (4)	
C15	0.35023 (13)	0.3678 (2)	0.71151 (8)	0.0479 (5)	
C16	0.36495 (13)	0.4871 (2)	0.73223 (8)	0.0481 (5)	
N17	0.35301 (12)	0.48713 (18)	0.78480 (7)	0.0516 (4)	
C17A	0.33304 (14)	0.3679 (2)	0.79516 (8)	0.0514 (5)	
C127	0.2866 (3)	0.0098 (3)	0.82642 (12)	0.1004 (12)	0.6289 (17)
H12A	0.3290	−0.0485	0.8154	0.121*	0.6289 (17)
H12B	0.2354	−0.0203	0.8126	0.121*	0.6289 (17)
C121	0.2822 (7)	0.009 (2)	0.88341 (16)	0.0601 (19)	0.6289 (17)
C122	0.2095 (4)	0.0462 (10)	0.90440 (18)	0.0712 (12)	0.6289 (17)
H122	0.1672	0.0698	0.8830	0.085*	0.6289 (17)
C123	0.1973 (4)	0.0495 (6)	0.95558 (18)	0.0795 (15)	0.6289 (17)
H123	0.1480	0.0776	0.9687	0.095*	0.6289 (17)
C124	0.2583 (4)	0.0110 (5)	0.98740 (18)	0.0750 (16)	0.6289 (17)
Cl14	0.23860 (15)	0.0201 (2)	1.05325 (5)	0.1395 (8)	0.6289 (17)
C125	0.3307 (5)	−0.0285 (9)	0.9695 (3)	0.098 (2)	0.6289 (17)
H125	0.3724	−0.0508	0.9916	0.118*	0.6289 (17)
C126	0.3419 (5)	−0.0352 (11)	0.9164 (2)	0.0913 (19)	0.6289 (17)
H126	0.3897	−0.0696	0.9034	0.110*	0.6289 (17)
C137	0.2866 (3)	0.0098 (3)	0.82642 (12)	0.1004 (12)	0.3711 (17)
H13A	0.2334	−0.0154	0.8141	0.121*	0.3711 (17)
H13B	0.3258	−0.0484	0.8115	0.121*	0.3711 (17)
C131	0.2878 (12)	−0.010 (4)	0.8818 (3)	0.0601 (19)	0.3711 (17)
C132	0.2312 (7)	0.0348 (19)	0.9149 (3)	0.0712 (12)	0.3711 (17)
H132	0.1853	0.0761	0.9023	0.085*	0.3711 (17)
C133	0.2404 (7)	0.0206 (11)	0.9664 (3)	0.0795 (15)	0.3711 (17)
H133	0.2012	0.0485	0.9892	0.095*	0.3711 (17)
C134	0.3110 (8)	−0.0370 (14)	0.9822 (3)	0.0750 (16)	0.3711 (17)
Cl34	0.3237 (3)	−0.0678 (4)	1.04645 (9)	0.1395 (8)	0.3711 (17)
C135	0.3710 (6)	−0.0735 (11)	0.9502 (4)	0.098 (2)	0.3711 (17)
H135	0.4177	−0.1122	0.9630	0.118*	0.3711 (17)
C136	0.3629 (8)	−0.053 (2)	0.8992 (4)	0.0913 (19)	0.3711 (17)
H136	0.4059	−0.0679	0.8770	0.110*	0.3711 (17)
C151	0.34906 (14)	0.3156 (2)	0.65887 (8)	0.0530 (5)	
H15A	0.3398	0.3856	0.6354	0.064*	
H15B	0.3040	0.2562	0.6557	0.064*	
N151	0.54541 (16)	0.1450 (2)	0.64974 (10)	0.0714 (6)	
H151	0.582 (2)	0.102 (3)	0.6632 (12)	0.086*	
C152	0.48211 (17)	0.1970 (3)	0.67633 (10)	0.0632 (6)	
H152	0.4780	0.1975	0.7115	0.076*	
C153	0.42624 (15)	0.2478 (2)	0.64434 (9)	0.0511 (5)	
C15A	0.45586 (16)	0.2239 (2)	0.59416 (9)	0.0556 (6)	
C154	0.4266 (2)	0.2512 (3)	0.54534 (10)	0.0778 (8)	
H154	0.3778	0.2946	0.5409	0.093*	
C155	0.4714 (3)	0.2129 (5)	0.50439 (13)	0.1129 (14)	
H155	0.4522	0.2300	0.4719	0.135*	
C156	0.5450 (3)	0.1491 (5)	0.51017 (15)	0.1260 (16)	
H156	0.5738	0.1243	0.4815	0.151*	
C157	0.5760 (2)	0.1219 (4)	0.55721 (15)	0.0997 (11)	
H157	0.6256	0.0800	0.5609	0.120*	
C15B	0.53082 (18)	0.1590 (3)	0.59925 (11)	0.0659 (7)	
C161	0.39381 (14)	0.6037 (2)	0.70655 (9)	0.0523 (5)	
C162	0.43854 (18)	0.5967 (3)	0.66191 (11)	0.0734 (8)	
H162	0.4498	0.5172	0.6476	0.088*	
C163	0.4663 (2)	0.7074 (4)	0.63880 (14)	0.0967 (11)	
H163	0.4949	0.7016	0.6085	0.116*	
C164	0.4523 (2)	0.8252 (3)	0.65985 (14)	0.0920 (10)	
H164	0.4714	0.8991	0.6440	0.110*	
C165	0.41005 (19)	0.8339 (3)	0.70429 (13)	0.0774 (8)	
H165	0.4013	0.9136	0.7191	0.093*	
C166	0.38022 (16)	0.7241 (2)	0.72725 (11)	0.0613 (6)	
H166	0.3505	0.7312	0.7571	0.074*	
S21	0.81319 (5)	0.26550 (6)	0.36890 (2)	0.06146 (19)	
C22	0.79667 (16)	0.1410 (2)	0.32528 (9)	0.0556 (6)	
N23	0.80388 (12)	0.16982 (18)	0.27773 (7)	0.0521 (5)	
N24	0.82302 (11)	0.29774 (17)	0.27438 (7)	0.0464 (4)	
C25	0.83901 (13)	0.3782 (2)	0.23401 (8)	0.0454 (5)	
C26	0.85977 (13)	0.4923 (2)	0.25691 (9)	0.0471 (5)	
N27	0.85510 (12)	0.48486 (17)	0.30993 (7)	0.0510 (4)	
C27A	0.83258 (14)	0.3658 (2)	0.31816 (8)	0.0488 (5)	
C227	0.7712 (2)	0.0096 (3)	0.34268 (10)	0.0756 (8)	
H27A	0.7934	−0.0536	0.3195	0.091*	
H27B	0.7124	0.0038	0.3409	0.091*	
C221	0.79792 (17)	−0.0234 (2)	0.39582 (9)	0.0581 (6)	
C222	0.7412 (2)	−0.0349 (3)	0.43409 (12)	0.0789 (8)	
H222	0.6865	−0.0214	0.4270	0.095*	
C223	0.7651 (2)	−0.0666 (3)	0.48308 (12)	0.0879 (10)	
H223	0.7265	−0.0758	0.5086	0.105*	
C224	0.8451 (2)	−0.0840 (3)	0.49320 (11)	0.0752 (8)	
Cl24	0.87453 (9)	−0.12121 (10)	0.55515 (3)	0.1245 (4)	
C225	0.9020 (2)	−0.0732 (3)	0.45637 (12)	0.0780 (8)	
H225	0.9567	−0.0858	0.4638	0.094*	
C226	0.87801 (19)	−0.0434 (3)	0.40794 (11)	0.0699 (7)	
H226	0.9171	−0.0366	0.3827	0.084*	
C251	0.82542 (14)	0.3397 (2)	0.18012 (8)	0.0495 (5)	
H25A	0.8160	0.4166	0.1602	0.059*	
H25B	0.7765	0.2880	0.1784	0.059*	
N251	1.00742 (14)	0.1509 (2)	0.14489 (9)	0.0629 (6)	
H251	1.0492 (18)	0.111 (3)	0.1531 (11)	0.075*	
C252	0.95708 (15)	0.2074 (2)	0.17995 (9)	0.0544 (6)	
H252	0.9652	0.2060	0.2148	0.065*	
C253	0.89371 (14)	0.2656 (2)	0.15627 (8)	0.0465 (5)	
C25A	0.90401 (16)	0.2437 (2)	0.10308 (9)	0.0514 (5)	
C254	0.8607 (2)	0.2795 (3)	0.05983 (10)	0.0694 (7)	
H254	0.8129	0.3268	0.0625	0.083*	
C255	0.8900 (3)	0.2433 (3)	0.01311 (11)	0.0889 (11)	
H255	0.8618	0.2675	−0.0159	0.107*	
C256	0.9603 (3)	0.1721 (3)	0.00835 (12)	0.0948 (12)	
H256	0.9782	0.1488	−0.0238	0.114*	
C257	1.0041 (2)	0.1350 (3)	0.04969 (12)	0.0810 (9)	
H257	1.0512	0.0867	0.0462	0.097*	
C25B	0.97612 (17)	0.1717 (2)	0.09748 (10)	0.0589 (6)	
C261	0.88792 (14)	0.6111 (2)	0.23229 (9)	0.0517 (5)	
C262	0.93528 (16)	0.6056 (3)	0.18874 (10)	0.0625 (6)	
H262	0.9476	0.5264	0.1744	0.075*	
C263	0.96429 (19)	0.7166 (3)	0.16649 (12)	0.0762 (8)	
H263	0.9953	0.7115	0.1371	0.091*	
C264	0.9476 (2)	0.8343 (3)	0.18741 (14)	0.0822 (9)	
H264	0.9676	0.9088	0.1725	0.099*	
C265	0.9009 (2)	0.8412 (3)	0.23078 (14)	0.0802 (9)	
H265	0.8896	0.9206	0.2452	0.096*	
C266	0.87069 (16)	0.7304 (2)	0.25296 (11)	0.0631 (7)	
H266	0.8387	0.7361	0.2819	0.076*	

### 2-(4-Chlorobenzyl)-5-[(1H-indol-3-yl)methyl]-6-phenylimidazo[2,1-b][1,3,4]thiadiazole (I) Atomic displacement parameters (Å^2^)

**Table:**

	*U*^11^	*U*^22^	*U*^33^	*U*^12^	*U*^13^	*U*^23^
S11	0.1014 (5)	0.0572 (4)	0.0434 (3)	−0.0198 (3)	0.0077 (3)	−0.0023 (3)
C12	0.094 (2)	0.0527 (14)	0.0504 (14)	−0.0186 (13)	0.0030 (13)	−0.0018 (11)
N13	0.0783 (14)	0.0487 (11)	0.0505 (11)	−0.0157 (10)	0.0016 (10)	−0.0024 (9)
N14	0.0599 (11)	0.0476 (10)	0.0425 (10)	−0.0103 (9)	0.0012 (8)	−0.0028 (8)
C15	0.0486 (12)	0.0510 (12)	0.0440 (11)	−0.0023 (10)	0.0021 (9)	0.0002 (9)
C16	0.0457 (11)	0.0505 (12)	0.0480 (12)	−0.0026 (9)	0.0028 (9)	0.0013 (9)
N17	0.0580 (11)	0.0484 (10)	0.0482 (10)	−0.0080 (9)	0.0040 (8)	−0.0035 (8)
C17A	0.0587 (13)	0.0510 (13)	0.0445 (12)	−0.0105 (10)	0.0028 (10)	−0.0044 (10)
C127	0.178 (4)	0.0584 (18)	0.0645 (19)	−0.034 (2)	0.018 (2)	0.0031 (14)
C121	0.086 (2)	0.035 (7)	0.0592 (15)	−0.0090 (16)	−0.0037 (14)	0.0072 (15)
C122	0.066 (4)	0.082 (3)	0.065 (3)	−0.003 (3)	−0.018 (2)	0.023 (3)
C123	0.087 (4)	0.095 (4)	0.056 (3)	−0.020 (3)	−0.001 (3)	0.024 (2)
C124	0.104 (5)	0.070 (3)	0.052 (2)	−0.031 (3)	−0.022 (3)	0.022 (2)
Cl14	0.213 (2)	0.1510 (16)	0.0543 (6)	−0.0869 (14)	−0.0184 (10)	0.0230 (8)
C125	0.115 (7)	0.078 (4)	0.102 (6)	0.000 (4)	−0.035 (4)	0.024 (4)
C126	0.076 (4)	0.076 (4)	0.121 (5)	0.015 (3)	0.005 (4)	0.022 (5)
C137	0.178 (4)	0.0584 (18)	0.0645 (19)	−0.034 (2)	0.018 (2)	0.0031 (14)
C131	0.086 (2)	0.035 (7)	0.0592 (15)	−0.0090 (16)	−0.0037 (14)	0.0072 (15)
C132	0.066 (4)	0.082 (3)	0.065 (3)	−0.003 (3)	−0.018 (2)	0.023 (3)
C133	0.087 (4)	0.095 (4)	0.056 (3)	−0.020 (3)	−0.001 (3)	0.024 (2)
C134	0.104 (5)	0.070 (3)	0.052 (2)	−0.031 (3)	−0.022 (3)	0.022 (2)
Cl34	0.213 (2)	0.1510 (16)	0.0543 (6)	−0.0869 (14)	−0.0184 (10)	0.0230 (8)
C135	0.115 (7)	0.078 (4)	0.102 (6)	0.000 (4)	−0.035 (4)	0.024 (4)
C136	0.076 (4)	0.076 (4)	0.121 (5)	0.015 (3)	0.005 (4)	0.022 (5)
C151	0.0577 (13)	0.0603 (14)	0.0409 (11)	−0.0030 (11)	−0.0025 (10)	−0.0014 (10)
N151	0.0752 (16)	0.0659 (14)	0.0733 (16)	0.0193 (12)	−0.0010 (12)	0.0093 (12)
C152	0.0718 (16)	0.0667 (16)	0.0511 (14)	0.0077 (13)	0.0001 (12)	0.0065 (12)
C153	0.0605 (14)	0.0484 (12)	0.0445 (12)	−0.0014 (10)	−0.0005 (10)	0.0005 (9)
C15A	0.0670 (15)	0.0509 (13)	0.0488 (13)	−0.0002 (11)	0.0047 (11)	0.0002 (10)
C154	0.094 (2)	0.093 (2)	0.0467 (14)	0.0082 (17)	0.0049 (14)	0.0025 (14)
C155	0.130 (3)	0.156 (4)	0.0524 (18)	0.025 (3)	0.015 (2)	−0.001 (2)
C156	0.142 (4)	0.161 (4)	0.075 (2)	0.040 (3)	0.035 (3)	−0.016 (3)
C157	0.100 (3)	0.105 (3)	0.094 (3)	0.031 (2)	0.024 (2)	−0.015 (2)
C15B	0.0782 (18)	0.0537 (14)	0.0657 (16)	0.0070 (13)	0.0097 (14)	−0.0016 (12)
C161	0.0487 (12)	0.0536 (13)	0.0546 (13)	−0.0038 (10)	−0.0002 (10)	0.0062 (10)
C162	0.0811 (19)	0.0667 (17)	0.0724 (18)	−0.0116 (14)	0.0222 (15)	0.0034 (14)
C163	0.116 (3)	0.092 (3)	0.082 (2)	−0.021 (2)	0.035 (2)	0.0145 (19)
C164	0.107 (3)	0.070 (2)	0.099 (3)	−0.0243 (18)	0.007 (2)	0.0288 (19)
C165	0.088 (2)	0.0534 (16)	0.091 (2)	−0.0062 (14)	−0.0023 (17)	0.0095 (15)
C166	0.0637 (15)	0.0519 (14)	0.0682 (16)	−0.0022 (11)	0.0015 (12)	0.0043 (12)
S21	0.0877 (5)	0.0533 (3)	0.0434 (3)	−0.0120 (3)	−0.0005 (3)	−0.0058 (2)
C22	0.0720 (16)	0.0473 (12)	0.0476 (13)	−0.0083 (11)	−0.0047 (11)	−0.0031 (10)
N23	0.0657 (12)	0.0418 (10)	0.0489 (11)	−0.0065 (9)	−0.0043 (9)	−0.0037 (8)
N24	0.0547 (10)	0.0406 (9)	0.0438 (10)	−0.0061 (8)	−0.0006 (8)	−0.0058 (7)
C25	0.0476 (12)	0.0429 (11)	0.0457 (11)	0.0008 (9)	−0.0009 (9)	−0.0029 (9)
C26	0.0456 (11)	0.0432 (11)	0.0524 (12)	0.0004 (9)	0.0006 (9)	−0.0034 (9)
N27	0.0597 (11)	0.0442 (10)	0.0490 (10)	−0.0041 (8)	−0.0002 (9)	−0.0089 (8)
C27A	0.0570 (13)	0.0468 (12)	0.0424 (11)	−0.0033 (10)	−0.0019 (9)	−0.0066 (9)
C227	0.114 (2)	0.0543 (15)	0.0585 (16)	−0.0228 (15)	−0.0099 (15)	0.0050 (12)
C221	0.0779 (17)	0.0439 (12)	0.0524 (13)	−0.0079 (11)	0.0025 (12)	−0.0008 (10)
C222	0.0716 (18)	0.089 (2)	0.0762 (19)	−0.0084 (16)	0.0064 (15)	0.0005 (16)
C223	0.110 (3)	0.093 (2)	0.0607 (18)	−0.013 (2)	0.0310 (18)	0.0006 (16)
C224	0.116 (3)	0.0518 (15)	0.0579 (16)	−0.0069 (16)	−0.0052 (17)	0.0053 (12)
Cl24	0.2214 (13)	0.0858 (6)	0.0662 (5)	−0.0033 (7)	−0.0249 (6)	0.0174 (4)
C225	0.089 (2)	0.0626 (17)	0.083 (2)	0.0063 (15)	−0.0084 (17)	0.0093 (15)
C226	0.0783 (19)	0.0632 (16)	0.0682 (17)	0.0017 (14)	0.0153 (14)	0.0066 (13)
C251	0.0540 (12)	0.0501 (12)	0.0446 (11)	0.0001 (10)	−0.0048 (9)	−0.0019 (9)
N251	0.0663 (13)	0.0551 (12)	0.0671 (14)	0.0146 (10)	0.0065 (11)	0.0045 (10)
C252	0.0621 (14)	0.0533 (13)	0.0480 (12)	0.0060 (11)	0.0009 (11)	0.0044 (10)
C253	0.0586 (13)	0.0399 (11)	0.0411 (11)	−0.0010 (9)	−0.0007 (9)	−0.0005 (8)
C25A	0.0741 (15)	0.0360 (11)	0.0442 (12)	−0.0035 (10)	0.0008 (11)	−0.0020 (9)
C254	0.105 (2)	0.0556 (15)	0.0473 (14)	−0.0033 (14)	−0.0139 (14)	−0.0021 (11)
C255	0.153 (3)	0.0666 (19)	0.0467 (15)	−0.014 (2)	−0.0099 (18)	−0.0047 (13)
C256	0.165 (4)	0.0663 (19)	0.0535 (17)	−0.023 (2)	0.025 (2)	−0.0167 (15)
C257	0.116 (3)	0.0515 (15)	0.075 (2)	−0.0059 (16)	0.0346 (18)	−0.0122 (14)
C25B	0.0802 (17)	0.0389 (12)	0.0576 (14)	−0.0028 (11)	0.0119 (12)	−0.0022 (10)
C261	0.0474 (12)	0.0454 (12)	0.0622 (14)	−0.0030 (10)	−0.0060 (10)	0.0011 (10)
C262	0.0636 (15)	0.0569 (14)	0.0669 (16)	−0.0066 (12)	0.0022 (12)	0.0014 (12)
C263	0.0725 (18)	0.076 (2)	0.080 (2)	−0.0145 (15)	0.0040 (15)	0.0146 (16)
C264	0.0770 (19)	0.0629 (18)	0.107 (3)	−0.0158 (15)	−0.0132 (18)	0.0284 (17)
C265	0.083 (2)	0.0448 (14)	0.113 (3)	−0.0007 (13)	−0.0102 (19)	0.0030 (15)
C266	0.0616 (15)	0.0455 (13)	0.0822 (18)	0.0015 (11)	0.0001 (13)	−0.0013 (12)

### 2-(4-Chlorobenzyl)-5-[(1H-indol-3-yl)methyl]-6-phenylimidazo[2,1-b][1,3,4]thiadiazole (I) Geometric parameters (Å, º)

**Table:**

S11—C17A	1.725 (2)	C164—C165	1.366 (5)
S11—C12	1.757 (3)	C164—H164	0.9300
C12—N13	1.293 (3)	C165—C166	1.384 (4)
C12—C127	1.505 (4)	C165—H165	0.9300
N13—N14	1.374 (3)	C166—H166	0.9300
N14—C17A	1.362 (3)	S21—C27A	1.728 (2)
N14—C15	1.380 (3)	S21—C22	1.755 (2)
C15—C16	1.379 (3)	C22—N23	1.296 (3)
C15—C151	1.492 (3)	C22—C227	1.504 (3)
C16—N17	1.401 (3)	N23—N24	1.372 (2)
C16—C161	1.471 (3)	N24—C27A	1.364 (3)
N17—C17A	1.314 (3)	N24—C25	1.381 (3)
C127—C121	1.506 (5)	C25—C26	1.376 (3)
C127—H12A	0.9700	C25—C251	1.494 (3)
C127—H12B	0.9700	C26—N27	1.403 (3)
C121—C122	1.373 (6)	C26—C261	1.473 (3)
C121—C126	1.392 (6)	N27—C27A	1.313 (3)
C122—C123	1.366 (6)	C227—C221	1.509 (4)
C122—H122	0.9300	C227—H27A	0.9700
C123—C124	1.368 (7)	C227—H27B	0.9700
C123—H123	0.9300	C221—C226	1.372 (4)
C124—C125	1.345 (10)	C221—C222	1.380 (4)
C124—Cl14	1.771 (5)	C222—C223	1.391 (4)
C125—C126	1.416 (8)	C222—H222	0.9300
C125—H125	0.9300	C223—C224	1.356 (5)
C126—H126	0.9300	C223—H223	0.9300
C131—C132	1.360 (9)	C224—C225	1.355 (5)
C131—C136	1.392 (8)	C224—Cl24	1.748 (3)
C132—C133	1.377 (8)	C225—C226	1.373 (4)
C132—H132	0.9300	C225—H225	0.9300
C133—C134	1.371 (11)	C226—H226	0.9300
C133—H133	0.9300	C251—C253	1.502 (3)
C134—C135	1.353 (13)	C251—H25A	0.9700
C134—Cl34	1.739 (8)	C251—H25B	0.9700
C135—C136	1.371 (10)	N251—C25B	1.370 (3)
C135—H135	0.9300	N251—C252	1.374 (3)
C136—H136	0.9300	N251—H251	0.83 (3)
C151—C153	1.503 (3)	C252—C253	1.358 (3)
C151—H15A	0.9700	C252—H252	0.9300
C151—H15B	0.9700	C253—C25A	1.432 (3)
N151—C15B	1.362 (4)	C25A—C254	1.397 (3)
N151—C152	1.368 (4)	C25A—C25B	1.411 (4)
N151—H151	0.82 (3)	C254—C255	1.377 (4)
C152—C153	1.355 (3)	C254—H254	0.9300
C152—H152	0.9300	C255—C256	1.380 (5)
C153—C15A	1.433 (3)	C255—H255	0.9300
C15A—C154	1.404 (4)	C256—C257	1.363 (5)
C15A—C15B	1.413 (4)	C256—H256	0.9300
C154—C155	1.368 (4)	C257—C25B	1.396 (4)
C154—H154	0.9300	C257—H257	0.9300
C155—C156	1.389 (6)	C261—C266	1.387 (3)
C155—H155	0.9300	C261—C262	1.390 (4)
C156—C157	1.372 (5)	C262—C263	1.382 (4)
C156—H156	0.9300	C262—H262	0.9300
C157—C15B	1.391 (4)	C263—C264	1.373 (5)
C157—H157	0.9300	C263—H263	0.9300
C161—C166	1.386 (4)	C264—C265	1.381 (5)
C161—C162	1.391 (4)	C264—H264	0.9300
C162—C163	1.382 (4)	C265—C266	1.386 (4)
C162—H162	0.9300	C265—H265	0.9300
C163—C164	1.367 (5)	C266—H266	0.9300
C163—H163	0.9300		
			
C17A—S11—C12	87.89 (12)	C165—C164—H164	120.2
N13—C12—C127	121.8 (2)	C163—C164—H164	120.2
N13—C12—S11	116.85 (19)	C164—C165—C166	120.1 (3)
C127—C12—S11	121.3 (2)	C164—C165—H165	119.9
C12—N13—N14	107.92 (19)	C166—C165—H165	119.9
C17A—N14—N13	118.21 (18)	C165—C166—C161	121.2 (3)
C17A—N14—C15	108.51 (19)	C165—C166—H166	119.4
N13—N14—C15	133.20 (19)	C161—C166—H166	119.4
C16—C15—N14	103.65 (19)	C27A—S21—C22	88.11 (11)
C16—C15—C151	134.4 (2)	N23—C22—C227	122.2 (2)
N14—C15—C151	121.9 (2)	N23—C22—S21	116.74 (18)
C15—C16—N17	111.61 (19)	C227—C22—S21	121.03 (18)
C15—C16—C161	128.2 (2)	C22—N23—N24	107.96 (18)
N17—C16—C161	120.1 (2)	C27A—N24—N23	118.44 (18)
C17A—N17—C16	103.93 (18)	C27A—N24—C25	108.43 (18)
N17—C17A—N14	112.3 (2)	N23—N24—C25	133.07 (18)
N17—C17A—S11	138.63 (18)	C26—C25—N24	103.45 (18)
N14—C17A—S11	109.09 (17)	C26—C25—C251	133.4 (2)
C12—C127—C121	111.2 (9)	N24—C25—C251	122.88 (19)
C12—C127—H12A	109.4	C25—C26—N27	112.13 (19)
C121—C127—H12A	109.4	C25—C26—C261	127.6 (2)
C12—C127—H12B	109.4	N27—C26—C261	120.22 (19)
C121—C127—H12B	109.4	C27A—N27—C26	103.44 (17)
H12A—C127—H12B	108.0	N27—C27A—N24	112.5 (2)
C122—C121—C126	117.1 (4)	N27—C27A—S21	138.73 (17)
C122—C121—C127	116.4 (5)	N24—C27A—S21	108.72 (16)
C126—C121—C127	126.3 (5)	C22—C227—C221	114.2 (2)
C123—C122—C121	122.3 (5)	C22—C227—H27A	108.7
C123—C122—H122	118.8	C221—C227—H27A	108.7
C121—C122—H122	118.8	C22—C227—H27B	108.7
C122—C123—C124	119.4 (5)	C221—C227—H27B	108.7
C122—C123—H123	120.3	H27A—C227—H27B	107.6
C124—C123—H123	120.3	C226—C221—C222	117.8 (3)
C125—C124—C123	121.6 (6)	C226—C221—C227	122.1 (3)
C125—C124—Cl14	121.6 (5)	C222—C221—C227	120.2 (3)
C123—C124—Cl14	116.9 (5)	C221—C222—C223	120.7 (3)
C124—C125—C126	118.6 (6)	C221—C222—H222	119.6
C124—C125—H125	120.7	C223—C222—H222	119.6
C126—C125—H125	120.7	C224—C223—C222	119.2 (3)
C121—C126—C125	120.7 (6)	C224—C223—H223	120.4
C121—C126—H126	119.7	C222—C223—H223	120.4
C125—C126—H126	119.7	C225—C224—C223	121.3 (3)
C132—C131—C136	120.7 (9)	C225—C224—Cl24	119.9 (3)
C131—C132—C133	121.4 (8)	C223—C224—Cl24	118.9 (3)
C131—C132—H132	119.3	C224—C225—C226	119.2 (3)
C133—C132—H132	119.3	C224—C225—H225	120.4
C134—C133—C132	116.1 (8)	C226—C225—H225	120.4
C134—C133—H133	122.0	C221—C226—C225	121.8 (3)
C132—C133—H133	122.0	C221—C226—H226	119.1
C135—C134—C133	123.5 (8)	C225—C226—H226	119.1
C135—C134—Cl34	117.9 (9)	C25—C251—C253	115.17 (18)
C133—C134—Cl34	118.5 (8)	C25—C251—H25A	108.5
C134—C135—C136	119.9 (9)	C253—C251—H25A	108.5
C134—C135—H135	120.0	C25—C251—H25B	108.5
C136—C135—H135	120.0	C253—C251—H25B	108.5
C135—C136—C131	117.2 (9)	H25A—C251—H25B	107.5
C135—C136—H136	121.4	C25B—N251—C252	108.7 (2)
C131—C136—H136	121.4	C25B—N251—H251	129 (2)
C15—C151—C153	113.48 (19)	C252—N251—H251	123 (2)
C15—C151—H15A	108.9	C253—C252—N251	110.1 (2)
C153—C151—H15A	108.9	C253—C252—H252	124.9
C15—C151—H15B	108.9	N251—C252—H252	124.9
C153—C151—H15B	108.9	C252—C253—C25A	106.8 (2)
H15A—C151—H15B	107.7	C252—C253—C251	127.7 (2)
C15B—N151—C152	109.0 (2)	C25A—C253—C251	125.6 (2)
C15B—N151—H151	127 (2)	C254—C25A—C25B	119.0 (2)
C152—N151—H151	123 (2)	C254—C25A—C253	134.3 (2)
C153—C152—N151	110.6 (2)	C25B—C25A—C253	106.7 (2)
C153—C152—H152	124.7	C255—C254—C25A	118.6 (3)
N151—C152—H152	124.7	C255—C254—H254	120.7
C152—C153—C15A	106.1 (2)	C25A—C254—H254	120.7
C152—C153—C151	126.7 (2)	C254—C255—C256	121.6 (3)
C15A—C153—C151	127.2 (2)	C254—C255—H255	119.2
C154—C15A—C15B	118.9 (2)	C256—C255—H255	119.2
C154—C15A—C153	134.1 (3)	C257—C256—C255	121.5 (3)
C15B—C15A—C153	107.0 (2)	C257—C256—H256	119.3
C155—C154—C15A	118.8 (3)	C255—C256—H256	119.3
C155—C154—H154	120.6	C256—C257—C25B	118.1 (3)
C15A—C154—H154	120.6	C256—C257—H257	120.9
C154—C155—C156	121.5 (4)	C25B—C257—H257	120.9
C154—C155—H155	119.2	N251—C25B—C257	131.1 (3)
C156—C155—H155	119.2	N251—C25B—C25A	107.7 (2)
C157—C156—C155	121.5 (3)	C257—C25B—C25A	121.2 (3)
C157—C156—H156	119.3	C266—C261—C262	118.5 (2)
C155—C156—H156	119.3	C266—C261—C26	121.0 (2)
C156—C157—C15B	117.7 (3)	C262—C261—C26	120.4 (2)
C156—C157—H157	121.1	C263—C262—C261	120.7 (3)
C15B—C157—H157	121.1	C263—C262—H262	119.7
N151—C15B—C157	131.0 (3)	C261—C262—H262	119.7
N151—C15B—C15A	107.3 (2)	C264—C263—C262	120.5 (3)
C157—C15B—C15A	121.6 (3)	C264—C263—H263	119.7
C166—C161—C162	117.8 (2)	C262—C263—H263	119.7
C166—C161—C16	120.9 (2)	C263—C264—C265	119.4 (3)
C162—C161—C16	121.2 (2)	C263—C264—H264	120.3
C163—C162—C161	120.3 (3)	C265—C264—H264	120.3
C163—C162—H162	119.9	C264—C265—C266	120.4 (3)
C161—C162—H162	119.9	C264—C265—H265	119.8
C164—C163—C162	121.0 (3)	C266—C265—H265	119.8
C164—C163—H163	119.5	C265—C266—C261	120.5 (3)
C162—C163—H163	119.5	C265—C266—H266	119.8
C165—C164—C163	119.6 (3)	C261—C266—H266	119.8
			
C17A—S11—C12—N13	−1.1 (2)	C163—C164—C165—C166	−1.4 (5)
C17A—S11—C12—C127	178.2 (3)	C164—C165—C166—C161	1.4 (5)
C127—C12—N13—N14	−179.3 (3)	C162—C161—C166—C165	0.1 (4)
S11—C12—N13—N14	0.0 (3)	C16—C161—C166—C165	177.4 (2)
C12—N13—N14—C17A	1.5 (3)	C27A—S21—C22—N23	−0.6 (2)
C12—N13—N14—C15	177.8 (3)	C27A—S21—C22—C227	−177.5 (2)
C17A—N14—C15—C16	1.7 (2)	C227—C22—N23—N24	176.4 (2)
N13—N14—C15—C16	−174.9 (2)	S21—C22—N23—N24	−0.5 (3)
C17A—N14—C15—C151	−176.6 (2)	C22—N23—N24—C27A	1.7 (3)
N13—N14—C15—C151	6.9 (4)	C22—N23—N24—C25	178.6 (2)
N14—C15—C16—N17	−1.8 (2)	C27A—N24—C25—C26	1.5 (2)
C151—C15—C16—N17	176.1 (2)	N23—N24—C25—C26	−175.6 (2)
N14—C15—C16—C161	175.0 (2)	C27A—N24—C25—C251	−173.0 (2)
C151—C15—C16—C161	−7.1 (4)	N23—N24—C25—C251	9.9 (4)
C15—C16—N17—C17A	1.3 (3)	N24—C25—C26—N27	−1.4 (2)
C161—C16—N17—C17A	−175.9 (2)	C251—C25—C26—N27	172.2 (2)
C16—N17—C17A—N14	−0.2 (3)	N24—C25—C26—C261	175.8 (2)
C16—N17—C17A—S11	177.7 (2)	C251—C25—C26—C261	−10.6 (4)
N13—N14—C17A—N17	176.1 (2)	C25—C26—N27—C27A	0.8 (2)
C15—N14—C17A—N17	−1.0 (3)	C261—C26—N27—C27A	−176.7 (2)
N13—N14—C17A—S11	−2.4 (3)	C26—N27—C27A—N24	0.2 (3)
C15—N14—C17A—S11	−179.52 (16)	C26—N27—C27A—S21	178.2 (2)
C12—S11—C17A—N17	−176.1 (3)	N23—N24—C27A—N27	176.49 (19)
C12—S11—C17A—N14	1.82 (19)	C25—N24—C27A—N27	−1.1 (3)
N13—C12—C127—C121	175.2 (4)	N23—N24—C27A—S21	−2.1 (3)
S11—C12—C127—C121	−4.1 (6)	C25—N24—C27A—S21	−179.69 (15)
C12—C127—C121—C122	82.4 (18)	C22—S21—C27A—N27	−176.7 (3)
C12—C127—C121—C126	−103.3 (19)	C22—S21—C27A—N24	1.38 (18)
C126—C121—C122—C123	5 (3)	N23—C22—C227—C221	156.4 (3)
C127—C121—C122—C123	179.8 (11)	S21—C22—C227—C221	−26.9 (4)
C121—C122—C123—C124	−2.1 (17)	C22—C227—C221—C226	−69.1 (4)
C122—C123—C124—C125	0.9 (10)	C22—C227—C221—C222	111.2 (3)
C122—C123—C124—Cl14	179.1 (6)	C226—C221—C222—C223	−0.4 (4)
C123—C124—C125—C126	−2.8 (11)	C227—C221—C222—C223	179.3 (3)
Cl14—C124—C125—C126	179.1 (7)	C221—C222—C223—C224	1.2 (5)
C122—C121—C126—C125	−7 (3)	C222—C223—C224—C225	−1.1 (5)
C127—C121—C126—C125	178.9 (15)	C222—C223—C224—Cl24	179.0 (3)
C124—C125—C126—C121	5.8 (18)	C223—C224—C225—C226	0.4 (5)
C136—C131—C132—C133	−11 (5)	Cl24—C224—C225—C226	−179.8 (2)
C131—C132—C133—C134	2 (3)	C222—C221—C226—C225	−0.4 (4)
C132—C133—C134—C135	3 (2)	C227—C221—C226—C225	179.9 (3)
C132—C133—C134—Cl34	−175.7 (12)	C224—C225—C226—C221	0.4 (5)
C133—C134—C135—C136	0 (2)	C26—C25—C251—C253	103.2 (3)
Cl34—C134—C135—C136	178.8 (14)	N24—C25—C251—C253	−84.2 (3)
C134—C135—C136—C131	−8 (3)	C25B—N251—C252—C253	−0.5 (3)
C132—C131—C136—C135	13 (5)	N251—C252—C253—C25A	0.6 (3)
C16—C15—C151—C153	98.6 (3)	N251—C252—C253—C251	−179.1 (2)
N14—C15—C151—C153	−83.8 (3)	C25—C251—C253—C252	14.9 (3)
C15B—N151—C152—C153	−1.2 (3)	C25—C251—C253—C25A	−164.8 (2)
N151—C152—C153—C15A	1.1 (3)	C252—C253—C25A—C254	−179.0 (3)
N151—C152—C153—C151	−178.8 (2)	C251—C253—C25A—C254	0.7 (4)
C15—C151—C153—C152	21.5 (4)	C252—C253—C25A—C25B	−0.5 (3)
C15—C151—C153—C15A	−158.3 (2)	C251—C253—C25A—C25B	179.2 (2)
C152—C153—C15A—C154	179.7 (3)	C25B—C25A—C254—C255	−0.1 (4)
C151—C153—C15A—C154	−0.5 (5)	C253—C25A—C254—C255	178.3 (3)
C152—C153—C15A—C15B	−0.6 (3)	C25A—C254—C255—C256	0.6 (5)
C151—C153—C15A—C15B	179.3 (2)	C254—C255—C256—C257	−0.5 (5)
C15B—C15A—C154—C155	0.6 (5)	C255—C256—C257—C25B	−0.3 (5)
C153—C15A—C154—C155	−179.6 (3)	C252—N251—C25B—C257	179.8 (3)
C15A—C154—C155—C156	−0.6 (7)	C252—N251—C25B—C25A	0.1 (3)
C154—C155—C156—C157	−0.1 (8)	C256—C257—C25B—N251	−178.8 (3)
C155—C156—C157—C15B	0.8 (7)	C256—C257—C25B—C25A	0.8 (4)
C152—N151—C15B—C157	−179.6 (3)	C254—C25A—C25B—N251	179.0 (2)
C152—N151—C15B—C15A	0.8 (3)	C253—C25A—C25B—N251	0.2 (3)
C156—C157—C15B—N151	179.7 (4)	C254—C25A—C25B—C257	−0.6 (4)
C156—C157—C15B—C15A	−0.7 (6)	C253—C25A—C25B—C257	−179.4 (2)
C154—C15A—C15B—N151	179.7 (3)	C25—C26—C261—C266	148.9 (2)
C153—C15A—C15B—N151	−0.1 (3)	N27—C26—C261—C266	−34.0 (3)
C154—C15A—C15B—C157	0.0 (4)	C25—C26—C261—C262	−33.7 (3)
C153—C15A—C15B—C157	−179.8 (3)	N27—C26—C261—C262	143.4 (2)
C15—C16—C161—C166	158.1 (2)	C266—C261—C262—C263	−0.2 (4)
N17—C16—C161—C166	−25.3 (3)	C26—C261—C262—C263	−177.7 (2)
C15—C16—C161—C162	−24.7 (4)	C261—C262—C263—C264	0.8 (4)
N17—C16—C161—C162	151.9 (2)	C262—C263—C264—C265	−0.5 (5)
C166—C161—C162—C163	−1.6 (4)	C263—C264—C265—C266	−0.3 (5)
C16—C161—C162—C163	−178.9 (3)	C264—C265—C266—C261	0.9 (4)
C161—C162—C163—C164	1.7 (6)	C262—C261—C266—C265	−0.6 (4)
C162—C163—C164—C165	−0.2 (6)	C26—C261—C266—C265	176.8 (2)

### 2-(4-Chlorobenzyl)-5-[(1H-indol-3-yl)methyl]-6-phenylimidazo[2,1-b][1,3,4]thiadiazole (I) Hydrogen-bond geometry (Å, º)

**Table:**

*D*—H···*A*	*D*—H	H···*A*	*D*···*A*	*D*—H···*A*
N151—H151···N17^i^	0.83 (3)	2.11 (3)	2.912 (3)	162 (3)
N251—H251···N27^ii^	0.83 (3)	2.27 (3)	3.087 (3)	167 (3)
C135—H135···*Cg*1^i^	0.93	2.52	3.272 (11)	138
C225—H225···*Cg*2^ii^	0.93	2.87	3.568 (4)	133
C252—H252···*Cg*3^ii^	0.93	2.77	3.568 (3)	144

Symmetry codes: (i) −*x*+1, *y*−1/2, −*z*+3/2; (ii) −*x*+2, *y*−1/2, −*z*+1/2.

### 2-(4-Chlorobenzyl)-6-(4-fluorophenyl)-5-[(1H-indol-3-yl)methyl]imidazo[2,1-b][1,3,4]thiadiazole (II) Crystal data

**Table:**

C_26_H_18_ClFN_4_S	*F*(000) = 976
*M**_r_* = 472.95	*D*_x_ = 1.370 Mg m^−^^3^
Monoclinic, *P*2_1_/*c*	Mo *K*α radiation, λ = 0.71073 Å
*a* = 15.340 (1) Å	Cell parameters from 5068 reflections
*b* = 11.1619 (7) Å	θ = 2.7–27.9°
*c* = 15.385 (1) Å	µ = 0.29 mm^−^^1^
β = 119.48 (1)°	*T* = 296 K
*V* = 2293.2 (3) Å^3^	Plate, yellow
*Z* = 4	0.46 × 0.44 × 0.20 mm

### 2-(4-Chlorobenzyl)-6-(4-fluorophenyl)-5-[(1H-indol-3-yl)methyl]imidazo[2,1-b][1,3,4]thiadiazole (II) Data collection

**Table:**

Oxford Diffraction Xcalibur with Sapphire CCD diffractometer	5041 independent reflections
Radiation source: Enhance (Mo) X-ray Source	3161 reflections with *I* > 2σ(*I*)
Graphite monochromator	*R*_int_ = 0.025
ω scans	θ_max_ = 27.5°, θ_min_ = 2.7°
Absorption correction: multi-scan (CrysAlis RED; Oxford Diffraction, 2009)	*h* = −19→19
*T*_min_ = 0.768, *T*_max_ = 0.944	*k* = −13→13
16612 measured reflections	*l* = −18→19

### 2-(4-Chlorobenzyl)-6-(4-fluorophenyl)-5-[(1H-indol-3-yl)methyl]imidazo[2,1-b][1,3,4]thiadiazole (II) Refinement

**Table:**

Refinement on *F*^2^	Primary atom site location: difference Fourier map
Least-squares matrix: full	Hydrogen site location: mixed
*R*[*F*^2^ > 2σ(*F*^2^)] = 0.050	H atoms treated by a mixture of independent and constrained refinement
*wR*(*F*^2^) = 0.131	*w* = 1/[σ^2^(*F*_o_^2^) + (0.052*P*)^2^ + 0.9215*P*] where *P* = (*F*_o_^2^ + 2*F*_c_^2^)/3
*S* = 1.01	(Δ/σ)_max_ < 0.001
5041 reflections	Δρ_max_ = 0.26 e Å^−^^3^
323 parameters	Δρ_min_ = −0.24 e Å^−^^3^
23 restraints	

### 2-(4-Chlorobenzyl)-6-(4-fluorophenyl)-5-[(1H-indol-3-yl)methyl]imidazo[2,1-b][1,3,4]thiadiazole (II) Special details

**Table:**

Geometry. All esds (except the esd in the dihedral angle between two l.s. planes) are estimated using the full covariance matrix. The cell esds are taken into account individually in the estimation of esds in distances, angles and torsion angles; correlations between esds in cell parameters are only used when they are defined by crystal symmetry. An approximate (isotropic) treatment of cell esds is used for estimating esds involving l.s. planes.

### 2-(4-Chlorobenzyl)-6-(4-fluorophenyl)-5-[(1H-indol-3-yl)methyl]imidazo[2,1-b][1,3,4]thiadiazole (II) Fractional atomic coordinates and isotropic or equivalent isotropic displacement parameters (Å^2^)

**Table:**

	*x*	*y*	*z*	*U*_iso_*/*U*_eq_	Occ. (<1)
S1	0.29020 (4)	0.26704 (6)	0.45226 (5)	0.0598 (2)	
C2	0.37289 (17)	0.1507 (2)	0.46549 (19)	0.0570 (6)	
N3	0.46687 (13)	0.17314 (16)	0.51939 (14)	0.0535 (5)	
N4	0.47701 (12)	0.28832 (15)	0.55296 (13)	0.0454 (4)	
C5	0.55903 (15)	0.35639 (19)	0.61424 (15)	0.0451 (5)	
C6	0.51834 (16)	0.46116 (19)	0.62492 (15)	0.0458 (5)	
N7	0.41374 (13)	0.45963 (16)	0.57021 (13)	0.0491 (4)	
C7A	0.39325 (15)	0.3533 (2)	0.52847 (16)	0.0474 (5)	
C27	0.33592 (19)	0.0335 (2)	0.4129 (3)	0.0840 (9)	0.822 (6)
H27A	0.3797	−0.0295	0.4554	0.101*	0.822 (6)
H27B	0.3408	0.0348	0.3524	0.101*	0.822 (6)
C21	0.2299 (2)	0.0027 (3)	0.3856 (3)	0.0569 (7)	0.822 (6)
C22	0.1533 (3)	0.0360 (6)	0.2954 (3)	0.0736 (11)	0.822 (6)
H22	0.1666	0.0816	0.2526	0.088*	0.822 (6)
C23	0.0569 (3)	0.0035 (7)	0.2665 (4)	0.0869 (12)	0.822 (6)
H23	0.0054	0.0241	0.2034	0.104*	0.822 (6)
C24	0.0368 (3)	−0.0584 (5)	0.3295 (4)	0.0727 (12)	0.822 (6)
Cl24	−0.08535 (12)	−0.1013 (2)	0.2922 (3)	0.1368 (11)	0.822 (6)
C25	0.1119 (3)	−0.0917 (4)	0.4210 (4)	0.0811 (12)	0.822 (6)
H25	0.0979	−0.1344	0.4646	0.097*	0.822 (6)
C26	0.2088 (3)	−0.0614 (5)	0.4481 (3)	0.0722 (12)	0.822 (6)
H26	0.2607	−0.0851	0.5101	0.087*	0.822 (6)
C37	0.33592 (19)	0.0335 (2)	0.4129 (3)	0.0840 (9)	0.178 (6)
H37A	0.3766	−0.0300	0.4576	0.101*	0.178 (6)
H37B	0.3460	0.0324	0.3555	0.101*	0.178 (6)
C31	0.2272 (6)	0.0060 (10)	0.3779 (12)	0.0569 (7)	0.178 (6)
C32	0.1626 (10)	0.034 (3)	0.2808 (14)	0.0736 (11)	0.178 (6)
H32	0.1874	0.0604	0.2399	0.088*	0.178 (6)
C33	0.0623 (10)	0.023 (4)	0.2433 (17)	0.0869 (12)	0.178 (6)
H33	0.0184	0.0567	0.1819	0.104*	0.178 (6)
C34	0.0261 (9)	−0.037 (3)	0.2944 (16)	0.0727 (12)	0.178 (6)
Cl34	−0.1018 (5)	−0.0635 (9)	0.2309 (12)	0.1368 (11)	0.178 (6)
C35	0.0864 (11)	−0.060 (2)	0.3928 (15)	0.0811 (12)	0.178 (6)
H35	0.0603	−0.0899	0.4317	0.097*	0.178 (6)
C36	0.1883 (10)	−0.037 (3)	0.4342 (15)	0.0722 (12)	0.178 (6)
H36	0.2306	−0.0521	0.5017	0.087*	0.178 (6)
C51	0.66292 (15)	0.3124 (2)	0.65056 (16)	0.0490 (5)	
H51A	0.7067	0.3806	0.6630	0.059*	
H51B	0.6642	0.2643	0.5987	0.059*	
N51	0.70786 (17)	0.11914 (19)	0.86245 (17)	0.0647 (6)	
H51	0.685 (2)	0.074 (3)	0.892 (2)	0.078*	
C52	0.64764 (17)	0.1730 (2)	0.77329 (18)	0.0552 (6)	
H52	0.5783	0.1655	0.7375	0.066*	
C53	0.70247 (15)	0.23931 (18)	0.74375 (16)	0.0454 (5)	
C53A	0.80430 (16)	0.22541 (19)	0.81938 (17)	0.0505 (5)	
C54	0.89531 (18)	0.2699 (2)	0.8328 (2)	0.0675 (7)	
H54	0.8971	0.3208	0.7858	0.081*	
C55	0.9821 (2)	0.2368 (3)	0.9171 (3)	0.0870 (10)	
H55	1.0432	0.2662	0.9273	0.104*	
C56	0.9801 (2)	0.1607 (3)	0.9869 (3)	0.0915 (10)	
H56	1.0401	0.1393	1.0428	0.110*	
C57	0.8927 (2)	0.1159 (3)	0.9764 (2)	0.0808 (9)	
H57	0.8923	0.0648	1.0240	0.097*	
C57A	0.80467 (18)	0.1494 (2)	0.89221 (18)	0.0581 (6)	
C61	0.57262 (17)	0.56426 (19)	0.68713 (17)	0.0488 (5)	
C62	0.66559 (18)	0.5491 (2)	0.77200 (18)	0.0600 (6)	
H62	0.6919	0.4723	0.7906	0.072*	
C63	0.7196 (2)	0.6456 (2)	0.8292 (2)	0.0684 (7)	
H63	0.7823	0.6348	0.8851	0.082*	
C64	0.6791 (2)	0.7564 (2)	0.8021 (2)	0.0660 (7)	
F64	0.73381 (13)	0.85184 (14)	0.85653 (14)	0.0915 (5)	
C65	0.5862 (2)	0.7756 (2)	0.7227 (2)	0.0687 (7)	
H65	0.5588	0.8522	0.7080	0.082*	
C66	0.53332 (19)	0.6786 (2)	0.66408 (19)	0.0582 (6)	
H66	0.4706	0.6907	0.6086	0.070*	

### 2-(4-Chlorobenzyl)-6-(4-fluorophenyl)-5-[(1H-indol-3-yl)methyl]imidazo[2,1-b][1,3,4]thiadiazole (II) Atomic displacement parameters (Å^2^)

**Table:**

	*U*^11^	*U*^22^	*U*^33^	*U*^12^	*U*^13^	*U*^23^
S1	0.0409 (3)	0.0612 (4)	0.0683 (4)	0.0045 (3)	0.0199 (3)	−0.0128 (3)
C2	0.0489 (13)	0.0515 (13)	0.0685 (15)	0.0016 (10)	0.0272 (12)	−0.0117 (12)
N3	0.0446 (10)	0.0464 (11)	0.0648 (12)	0.0021 (8)	0.0234 (9)	−0.0089 (9)
N4	0.0415 (9)	0.0444 (10)	0.0483 (10)	0.0047 (8)	0.0205 (8)	−0.0039 (8)
C5	0.0462 (11)	0.0457 (12)	0.0417 (11)	0.0006 (9)	0.0202 (10)	0.0016 (9)
C6	0.0496 (12)	0.0450 (12)	0.0434 (12)	0.0024 (10)	0.0233 (10)	0.0029 (10)
N7	0.0499 (11)	0.0460 (11)	0.0505 (11)	0.0056 (8)	0.0241 (9)	−0.0014 (9)
C7A	0.0436 (11)	0.0510 (13)	0.0463 (12)	0.0066 (10)	0.0211 (10)	−0.0002 (10)
C27	0.0577 (16)	0.0643 (17)	0.127 (3)	−0.0081 (13)	0.0428 (17)	−0.0350 (17)
C21	0.0526 (14)	0.0478 (13)	0.0664 (17)	−0.0039 (11)	0.0264 (13)	−0.0159 (12)
C22	0.0705 (19)	0.100 (2)	0.057 (2)	−0.0097 (18)	0.0358 (16)	0.0005 (19)
C23	0.069 (2)	0.107 (4)	0.056 (3)	−0.004 (2)	0.0089 (18)	0.001 (2)
C24	0.0492 (17)	0.064 (3)	0.097 (4)	−0.0151 (18)	0.030 (2)	−0.018 (3)
Cl24	0.0644 (7)	0.1099 (12)	0.221 (3)	−0.0290 (7)	0.0590 (12)	−0.0207 (14)
C25	0.090 (3)	0.066 (3)	0.099 (3)	−0.005 (2)	0.056 (3)	0.020 (2)
C26	0.068 (2)	0.064 (3)	0.064 (2)	0.008 (2)	0.0168 (18)	0.0131 (16)
C37	0.0577 (16)	0.0643 (17)	0.127 (3)	−0.0081 (13)	0.0428 (17)	−0.0350 (17)
C31	0.0526 (14)	0.0478 (13)	0.0664 (17)	−0.0039 (11)	0.0264 (13)	−0.0159 (12)
C32	0.0705 (19)	0.100 (2)	0.057 (2)	−0.0097 (18)	0.0358 (16)	0.0005 (19)
C33	0.069 (2)	0.107 (4)	0.056 (3)	−0.004 (2)	0.0089 (18)	0.001 (2)
C34	0.0492 (17)	0.064 (3)	0.097 (4)	−0.0151 (18)	0.030 (2)	−0.018 (3)
Cl34	0.0644 (7)	0.1099 (12)	0.221 (3)	−0.0290 (7)	0.0590 (12)	−0.0207 (14)
C35	0.090 (3)	0.066 (3)	0.099 (3)	−0.005 (2)	0.056 (3)	0.020 (2)
C36	0.068 (2)	0.064 (3)	0.064 (2)	0.008 (2)	0.0168 (18)	0.0131 (16)
C51	0.0426 (11)	0.0523 (13)	0.0526 (13)	−0.0012 (10)	0.0237 (10)	−0.0027 (10)
N51	0.0671 (14)	0.0548 (13)	0.0692 (14)	−0.0010 (10)	0.0313 (12)	0.0142 (11)
C52	0.0492 (13)	0.0519 (13)	0.0596 (15)	−0.0005 (11)	0.0230 (12)	0.0035 (11)
C53	0.0407 (11)	0.0408 (12)	0.0533 (13)	0.0018 (9)	0.0221 (10)	−0.0020 (10)
C53A	0.0463 (12)	0.0391 (12)	0.0608 (14)	0.0049 (9)	0.0223 (11)	−0.0029 (11)
C54	0.0469 (13)	0.0648 (16)	0.0834 (18)	−0.0001 (12)	0.0265 (13)	−0.0016 (14)
C55	0.0450 (14)	0.086 (2)	0.108 (3)	0.0014 (14)	0.0204 (16)	−0.011 (2)
C56	0.0654 (19)	0.078 (2)	0.088 (2)	0.0222 (16)	0.0051 (17)	0.0004 (18)
C57	0.078 (2)	0.0622 (17)	0.0742 (19)	0.0153 (15)	0.0164 (16)	0.0118 (14)
C57A	0.0590 (15)	0.0406 (13)	0.0632 (15)	0.0081 (11)	0.0213 (12)	0.0019 (11)
C61	0.0595 (14)	0.0441 (12)	0.0518 (13)	−0.0012 (10)	0.0343 (12)	−0.0004 (10)
C62	0.0647 (15)	0.0517 (14)	0.0577 (14)	−0.0010 (12)	0.0255 (13)	−0.0039 (12)
C63	0.0678 (16)	0.0641 (17)	0.0685 (17)	−0.0094 (13)	0.0298 (14)	−0.0132 (14)
C64	0.0787 (18)	0.0556 (16)	0.0814 (18)	−0.0219 (14)	0.0530 (16)	−0.0191 (14)
F64	0.1081 (13)	0.0656 (10)	0.1202 (14)	−0.0371 (9)	0.0711 (11)	−0.0366 (9)
C65	0.089 (2)	0.0401 (13)	0.098 (2)	−0.0016 (13)	0.0619 (18)	−0.0014 (14)
C66	0.0650 (15)	0.0508 (14)	0.0680 (16)	0.0028 (11)	0.0399 (13)	0.0035 (12)

### 2-(4-Chlorobenzyl)-6-(4-fluorophenyl)-5-[(1H-indol-3-yl)methyl]imidazo[2,1-b][1,3,4]thiadiazole (II) Geometric parameters (Å, º)

**Table:**

S1—C7A	1.725 (2)	C35—C36	1.390 (9)
S1—C2	1.756 (2)	C35—H35	0.9300
C2—N3	1.285 (3)	C36—H36	0.9300
C2—C27	1.495 (3)	C51—C53	1.494 (3)
N3—N4	1.365 (2)	C51—H51A	0.9700
N4—C7A	1.357 (3)	C51—H51B	0.9700
N4—C5	1.372 (3)	N51—C52	1.360 (3)
C5—C6	1.373 (3)	N51—C57A	1.364 (3)
C5—C51	1.489 (3)	N51—H51	0.86 (3)
C6—N7	1.398 (3)	C52—C53	1.355 (3)
C6—C61	1.466 (3)	C52—H52	0.9300
N7—C7A	1.312 (3)	C53—C53A	1.424 (3)
C27—C21	1.506 (4)	C53A—C54	1.398 (3)
C27—H27A	0.9700	C53A—C57A	1.403 (3)
C27—H27B	0.9700	C54—C55	1.376 (4)
C21—C22	1.356 (4)	C54—H54	0.9300
C21—C26	1.359 (4)	C55—C56	1.383 (5)
C22—C23	1.368 (5)	C55—H55	0.9300
C22—H22	0.9300	C56—C57	1.363 (4)
C23—C24	1.343 (5)	C56—H56	0.9300
C23—H23	0.9300	C57—C57A	1.386 (3)
C24—C25	1.361 (5)	C57—H57	0.9300
C24—Cl24	1.735 (3)	C61—C66	1.381 (3)
C25—C26	1.374 (5)	C61—C62	1.391 (3)
C25—H25	0.9300	C62—C63	1.380 (3)
C26—H26	0.9300	C62—H62	0.9300
C31—C36	1.360 (9)	C63—C64	1.355 (4)
C31—C32	1.361 (9)	C63—H63	0.9300
C32—C33	1.358 (9)	C64—F64	1.360 (3)
C32—H32	0.9300	C64—C65	1.363 (4)
C33—C34	1.343 (10)	C65—C66	1.387 (3)
C33—H33	0.9300	C65—H65	0.9300
C34—C35	1.353 (10)	C66—H66	0.9300
C34—Cl34	1.733 (8)		
			
C7A—S1—C2	88.03 (11)	C31—C36—C35	121.3 (11)
N3—C2—C27	121.7 (2)	C31—C36—H36	119.3
N3—C2—S1	116.61 (17)	C35—C36—H36	119.3
C27—C2—S1	121.61 (18)	C5—C51—C53	113.22 (18)
C2—N3—N4	108.07 (17)	C5—C51—H51A	108.9
C7A—N4—N3	118.80 (17)	C53—C51—H51A	108.9
C7A—N4—C5	108.52 (17)	C5—C51—H51B	108.9
N3—N4—C5	132.59 (17)	C53—C51—H51B	108.9
N4—C5—C6	103.70 (18)	H51A—C51—H51B	107.7
N4—C5—C51	121.71 (19)	C52—N51—C57A	108.4 (2)
C6—C5—C51	134.6 (2)	C52—N51—H51	123.0 (18)
C5—C6—N7	111.86 (18)	C57A—N51—H51	128.6 (18)
C5—C6—C61	126.9 (2)	C53—C52—N51	110.7 (2)
N7—C6—C61	121.16 (19)	C53—C52—H52	124.6
C7A—N7—C6	103.48 (17)	N51—C52—H52	124.6
N7—C7A—N4	112.42 (18)	C52—C53—C53A	106.2 (2)
N7—C7A—S1	139.12 (17)	C52—C53—C51	126.42 (19)
N4—C7A—S1	108.44 (15)	C53A—C53—C51	127.4 (2)
C2—C27—C21	114.9 (2)	C54—C53A—C57A	119.1 (2)
C2—C27—H27A	108.5	C54—C53A—C53	134.1 (2)
C21—C27—H27A	108.5	C57A—C53A—C53	106.9 (2)
C2—C27—H27B	108.5	C55—C54—C53A	118.4 (3)
C21—C27—H27B	108.5	C55—C54—H54	120.8
H27A—C27—H27B	107.5	C53A—C54—H54	120.8
C22—C21—C26	118.8 (3)	C54—C55—C56	121.1 (3)
C22—C21—C27	119.7 (3)	C54—C55—H55	119.5
C26—C21—C27	121.5 (3)	C56—C55—H55	119.5
C21—C22—C23	120.8 (4)	C57—C56—C55	122.0 (3)
C21—C22—H22	119.6	C57—C56—H56	119.0
C23—C22—H22	119.6	C55—C56—H56	119.0
C24—C23—C22	119.9 (3)	C56—C57—C57A	117.4 (3)
C24—C23—H23	120.0	C56—C57—H57	121.3
C22—C23—H23	120.0	C57A—C57—H57	121.3
C23—C24—C25	120.5 (3)	N51—C57A—C57	130.3 (3)
C23—C24—Cl24	119.8 (3)	N51—C57A—C53A	107.8 (2)
C25—C24—Cl24	119.6 (3)	C57—C57A—C53A	121.9 (3)
C24—C25—C26	119.0 (3)	C66—C61—C62	118.0 (2)
C24—C25—H25	120.5	C66—C61—C6	121.5 (2)
C26—C25—H25	120.5	C62—C61—C6	120.5 (2)
C21—C26—C25	120.9 (3)	C63—C62—C61	121.4 (2)
C21—C26—H26	119.6	C63—C62—H62	119.3
C25—C26—H26	119.6	C61—C62—H62	119.3
C36—C31—C32	118.0 (9)	C64—C63—C62	118.4 (3)
C33—C32—C31	120.4 (11)	C64—C63—H63	120.8
C33—C32—H32	119.8	C62—C63—H63	120.8
C31—C32—H32	119.8	C63—C64—F64	118.4 (3)
C34—C33—C32	120.4 (12)	C63—C64—C65	122.6 (2)
C34—C33—H33	119.8	F64—C64—C65	119.0 (2)
C32—C33—H33	119.8	C64—C65—C66	118.6 (2)
C33—C34—C35	119.7 (10)	C64—C65—H65	120.7
C33—C34—Cl34	115.7 (11)	C66—C65—H65	120.7
C35—C34—Cl34	124.1 (11)	C61—C66—C65	120.9 (2)
C34—C35—C36	118.3 (11)	C61—C66—H66	119.6
C34—C35—H35	120.8	C65—C66—H66	119.6
C36—C35—H35	120.8		
			
C7A—S1—C2—N3	−0.8 (2)	C33—C34—C35—C36	−10 (5)
C7A—S1—C2—C27	−178.1 (3)	Cl34—C34—C35—C36	179 (2)
C27—C2—N3—N4	176.9 (2)	C32—C31—C36—C35	4 (3)
S1—C2—N3—N4	−0.4 (3)	C34—C35—C36—C31	0 (4)
C2—N3—N4—C7A	1.9 (3)	N4—C5—C51—C53	−86.5 (2)
C2—N3—N4—C5	178.1 (2)	C6—C5—C51—C53	95.9 (3)
C7A—N4—C5—C6	1.1 (2)	C57A—N51—C52—C53	−0.6 (3)
N3—N4—C5—C6	−175.4 (2)	N51—C52—C53—C53A	0.4 (3)
C7A—N4—C5—C51	−177.14 (19)	N51—C52—C53—C51	−179.3 (2)
N3—N4—C5—C51	6.3 (4)	C5—C51—C53—C52	27.7 (3)
N4—C5—C6—N7	−1.2 (2)	C5—C51—C53—C53A	−151.9 (2)
C51—C5—C6—N7	176.8 (2)	C52—C53—C53A—C54	−180.0 (3)
N4—C5—C6—C61	176.9 (2)	C51—C53—C53A—C54	−0.3 (4)
C51—C5—C6—C61	−5.2 (4)	C52—C53—C53A—C57A	−0.1 (2)
C5—C6—N7—C7A	0.7 (2)	C51—C53—C53A—C57A	179.6 (2)
C61—C6—N7—C7A	−177.5 (2)	C57A—C53A—C54—C55	0.3 (4)
C6—N7—C7A—N4	0.0 (2)	C53—C53A—C54—C55	−179.8 (2)
C6—N7—C7A—S1	178.2 (2)	C53A—C54—C55—C56	0.4 (4)
N3—N4—C7A—N7	176.32 (19)	C54—C55—C56—C57	−0.7 (5)
C5—N4—C7A—N7	−0.8 (3)	C55—C56—C57—C57A	0.1 (5)
N3—N4—C7A—S1	−2.4 (2)	C52—N51—C57A—C57	−178.9 (3)
C5—N4—C7A—S1	−179.50 (14)	C52—N51—C57A—C53A	0.5 (3)
C2—S1—C7A—N7	−176.6 (3)	C56—C57—C57A—N51	180.0 (3)
C2—S1—C7A—N4	1.65 (17)	C56—C57—C57A—C53A	0.7 (4)
N3—C2—C27—C21	158.5 (3)	C54—C53A—C57A—N51	179.7 (2)
S1—C2—C27—C21	−24.3 (4)	C53—C53A—C57A—N51	−0.2 (3)
C2—C27—C21—C22	90.8 (5)	C54—C53A—C57A—C57	−0.9 (4)
C2—C27—C21—C26	−91.3 (5)	C53—C53A—C57A—C57	179.2 (2)
C26—C21—C22—C23	−1.6 (7)	C5—C6—C61—C66	153.4 (2)
C27—C21—C22—C23	176.4 (5)	N7—C6—C61—C66	−28.7 (3)
C21—C22—C23—C24	2.6 (9)	C5—C6—C61—C62	−27.0 (4)
C22—C23—C24—C25	−1.6 (10)	N7—C6—C61—C62	150.8 (2)
C22—C23—C24—Cl24	−179.2 (5)	C66—C61—C62—C63	−3.0 (4)
C23—C24—C25—C26	−0.2 (9)	C6—C61—C62—C63	177.3 (2)
Cl24—C24—C25—C26	177.4 (4)	C61—C62—C63—C64	1.3 (4)
C22—C21—C26—C25	−0.3 (7)	C62—C63—C64—F64	−177.8 (2)
C27—C21—C26—C25	−178.2 (4)	C62—C63—C64—C65	2.1 (4)
C24—C25—C26—C21	1.1 (8)	C63—C64—C65—C66	−3.6 (4)
C36—C31—C32—C33	3 (3)	F64—C64—C65—C66	176.3 (2)
C31—C32—C33—C34	−13 (5)	C62—C61—C66—C65	1.5 (3)
C32—C33—C34—C35	17 (6)	C6—C61—C66—C65	−178.9 (2)
C32—C33—C34—Cl34	−171 (2)	C64—C65—C66—C61	1.8 (4)

### 2-(4-Chlorobenzyl)-6-(4-fluorophenyl)-5-[(1H-indol-3-yl)methyl]imidazo[2,1-b][1,3,4]thiadiazole (II) Hydrogen-bond geometry (Å, º)

**Table:**

*D*—H···*A*	*D*—H	H···*A*	*D*···*A*	*D*—H···*A*
N51—H51···N7^i^	0.86 (3)	2.27 (3)	3.102 (3)	165 (3)
C25—H25···*Cg*1^i^	0.93	2.75	3.637 (5)	161
C35—H35···*Cg*1^i^	0.93	3.32	4.062 (5)	139

Symmetry code: (i) −*x*+1, *y*−1/2, −*z*+3/2.

### 6-(4-Bromophenyl)-2-(4-chlorobenzyl)-5-[(1H-indol-3-yl)methyl]imidazo[2,1-b][1,3,4]thiadiazole (III) Crystal data

**Table:**

C_26_H_18_BrClN_4_S	*D*_x_ = 1.491 Mg m^−^^3^
*M**_r_* = 533.85	Mo *K*α radiation, λ = 0.71073 Å
Orthorhombic, *P*2_1_2_1_2_1_	Cell parameters from 4645 reflections
*a* = 9.5735 (8) Å	θ = 2.6–27.9°
*b* = 9.6860 (9) Å	µ = 1.95 mm^−^^1^
*c* = 25.644 (2) Å	*T* = 296 K
*V* = 2377.9 (4) Å^3^	Block, yellow
*Z* = 4	0.48 × 0.44 × 0.44 mm
*F*(000) = 1080	

### 6-(4-Bromophenyl)-2-(4-chlorobenzyl)-5-[(1H-indol-3-yl)methyl]imidazo[2,1-b][1,3,4]thiadiazole (III) Data collection

**Table:**

Oxford Diffraction Xcalibur with Sapphire CCD diffractometer	4645 independent reflections
Radiation source: Enhance (Mo) X-ray Source	3140 reflections with *I* > 2σ(*I*)
Graphite monochromator	*R*_int_ = 0.030
ω scans	θ_max_ = 27.9°, θ_min_ = 2.6°
Absorption correction: multi-scan (CrysAlis RED; Oxford Diffraction, 2009)	*h* = −11→12
*T*_min_ = 0.368, *T*_max_ = 0.424	*k* = −11→12
10501 measured reflections	*l* = −32→27

### 6-(4-Bromophenyl)-2-(4-chlorobenzyl)-5-[(1H-indol-3-yl)methyl]imidazo[2,1-b][1,3,4]thiadiazole (III) Refinement

**Table:**

Refinement on *F*^2^	Hydrogen site location: mixed
Least-squares matrix: full	H atoms treated by a mixture of independent and constrained refinement
*R*[*F*^2^ > 2σ(*F*^2^)] = 0.045	*w* = 1/[σ^2^(*F*_o_^2^) + (0.0488*P*)^2^ + 0.2472*P*] where *P* = (*F*_o_^2^ + 2*F*_c_^2^)/3
*wR*(*F*^2^) = 0.100	(Δ/σ)_max_ < 0.001
*S* = 1.02	Δρ_max_ = 0.40 e Å^−^^3^
4645 reflections	Δρ_min_ = −0.51 e Å^−^^3^
323 parameters	Absolute structure: Flack *x* determined using 943 quotients [(*I*^+^)-(*I*^-^)]/[(*I*^+^)+(*I*^-^)] (Parsons *et al.*, 2013)
18 restraints	Absolute structure parameter: 0.014 (5)
Primary atom site location: difference Fourier map	

### 6-(4-Bromophenyl)-2-(4-chlorobenzyl)-5-[(1H-indol-3-yl)methyl]imidazo[2,1-b][1,3,4]thiadiazole (III) Special details

**Table:**

Geometry. All esds (except the esd in the dihedral angle between two l.s. planes) are estimated using the full covariance matrix. The cell esds are taken into account individually in the estimation of esds in distances, angles and torsion angles; correlations between esds in cell parameters are only used when they are defined by crystal symmetry. An approximate (isotropic) treatment of cell esds is used for estimating esds involving l.s. planes.

### 6-(4-Bromophenyl)-2-(4-chlorobenzyl)-5-[(1H-indol-3-yl)methyl]imidazo[2,1-b][1,3,4]thiadiazole (III) Fractional atomic coordinates and isotropic or equivalent isotropic displacement parameters (Å^2^)

**Table:**

	*x*	*y*	*z*	*U*_iso_*/*U*_eq_	Occ. (<1)
S1	0.83700 (15)	0.41040 (12)	0.58869 (4)	0.0607 (4)	
C2	0.8115 (5)	0.5817 (5)	0.60822 (16)	0.0521 (11)	
N3	0.7295 (4)	0.6558 (4)	0.58024 (14)	0.0484 (9)	
N4	0.6814 (4)	0.5756 (3)	0.53971 (12)	0.0420 (8)	
C5	0.5916 (4)	0.5991 (4)	0.49850 (14)	0.0398 (9)	
C6	0.5915 (5)	0.4749 (4)	0.47243 (16)	0.0421 (10)	
N7	0.6775 (4)	0.3777 (3)	0.49620 (12)	0.0463 (8)	
C7A	0.7273 (5)	0.4426 (4)	0.53667 (16)	0.0442 (11)	
C27	0.8805 (6)	0.6365 (6)	0.65587 (17)	0.0659 (14)	0.839 (5)
H27A	0.8988	0.7342	0.6512	0.079*	0.839 (5)
H27B	0.9695	0.5903	0.6606	0.079*	0.839 (5)
C21	0.792 (3)	0.6161 (7)	0.7041 (7)	0.0583 (16)	0.839 (5)
C22	0.8012 (8)	0.4946 (7)	0.7315 (2)	0.072 (2)	0.839 (5)
H22	0.8610	0.4253	0.7201	0.086*	0.839 (5)
C23	0.7217 (8)	0.4742 (8)	0.7761 (2)	0.078 (2)	0.839 (5)
H23	0.7263	0.3912	0.7942	0.094*	0.839 (5)
C24	0.6363 (7)	0.5786 (10)	0.7928 (3)	0.072 (2)	0.839 (5)
Cl24	0.5446 (3)	0.5544 (4)	0.85097 (8)	0.1052 (12)	0.839 (5)
C25	0.6247 (11)	0.6984 (10)	0.7669 (4)	0.093 (3)	0.839 (5)
H25	0.5661	0.7679	0.7790	0.111*	0.839 (5)
C26	0.7030 (9)	0.7164 (8)	0.7212 (4)	0.081 (3)	0.839 (5)
H26	0.6940	0.7978	0.7023	0.097*	0.839 (5)
C37	0.8805 (6)	0.6365 (6)	0.65587 (17)	0.0659 (14)	0.161 (5)
H37A	0.9104	0.7306	0.6494	0.079*	0.161 (5)
H37B	0.9631	0.5818	0.6632	0.079*	0.161 (5)
C31	0.786 (13)	0.635 (3)	0.703 (4)	0.0583 (16)	0.161 (5)
C32	0.733 (4)	0.515 (3)	0.7245 (12)	0.072 (2)	0.161 (5)
H32	0.7558	0.4307	0.7096	0.086*	0.161 (5)
C33	0.646 (4)	0.519 (3)	0.7682 (12)	0.078 (2)	0.161 (5)
H33	0.6213	0.4386	0.7853	0.094*	0.161 (5)
C34	0.599 (6)	0.645 (3)	0.7853 (16)	0.072 (2)	0.161 (5)
Cl34	0.4934 (14)	0.653 (2)	0.8408 (5)	0.1052 (12)	0.161 (5)
C35	0.644 (6)	0.763 (3)	0.7640 (19)	0.093 (3)	0.161 (5)
H35	0.6135	0.8473	0.7768	0.111*	0.161 (5)
C36	0.739 (6)	0.758 (3)	0.7223 (18)	0.081 (3)	0.161 (5)
H36	0.7694	0.8398	0.7074	0.097*	0.161 (5)
C51	0.5177 (5)	0.7330 (4)	0.49086 (18)	0.0491 (11)	
H51A	0.4648	0.7523	0.5223	0.059*	
H51B	0.4508	0.7209	0.4628	0.059*	
N51	0.6774 (5)	1.0775 (4)	0.47897 (15)	0.0592 (10)	
H51	0.669 (5)	1.175 (5)	0.4903 (17)	0.071*	
C52	0.5912 (5)	0.9832 (5)	0.50168 (18)	0.0517 (11)	
H52	0.5316	1.0024	0.5294	0.062*	
C53	0.6035 (4)	0.8576 (4)	0.47868 (16)	0.0441 (11)	
C53A	0.7043 (5)	0.8739 (5)	0.43823 (15)	0.0455 (10)	
C54	0.7624 (5)	0.7861 (5)	0.40072 (18)	0.0572 (12)	
H54	0.7335	0.6946	0.3982	0.069*	
C55	0.8628 (6)	0.8371 (6)	0.3677 (2)	0.0751 (16)	
H55	0.9015	0.7791	0.3427	0.090*	
C56	0.9083 (7)	0.9735 (7)	0.3707 (2)	0.0797 (18)	
H56	0.9777	1.0046	0.3482	0.096*	
C57	0.8518 (6)	1.0620 (6)	0.4065 (2)	0.0687 (15)	
H57	0.8813	1.1534	0.4084	0.082*	
C57A	0.7497 (5)	1.0127 (5)	0.43978 (18)	0.0523 (12)	
C61	0.5199 (4)	0.4425 (4)	0.42318 (15)	0.0407 (10)	
C62	0.3870 (5)	0.4889 (5)	0.41314 (17)	0.0540 (12)	
H62	0.3377	0.5345	0.4392	0.065*	
C63	0.3253 (5)	0.4693 (5)	0.36521 (18)	0.0591 (13)	
H63	0.2361	0.5032	0.3587	0.071*	
C64	0.3972 (5)	0.3988 (6)	0.32711 (16)	0.0573 (13)	
Br64	0.31651 (7)	0.38367 (7)	0.25942 (2)	0.0898 (3)	
C65	0.5256 (5)	0.3449 (5)	0.33654 (17)	0.0593 (13)	
H65	0.5717	0.2937	0.3111	0.071*	
C66	0.5865 (5)	0.3678 (5)	0.38478 (16)	0.0524 (12)	
H66	0.6748	0.3318	0.3914	0.063*	

### 6-(4-Bromophenyl)-2-(4-chlorobenzyl)-5-[(1H-indol-3-yl)methyl]imidazo[2,1-b][1,3,4]thiadiazole (III) Atomic displacement parameters (Å^2^)

**Table:**

	*U*^11^	*U*^22^	*U*^33^	*U*^12^	*U*^13^	*U*^23^
S1	0.0771 (8)	0.0558 (7)	0.0490 (6)	0.0067 (8)	−0.0131 (6)	0.0130 (6)
C2	0.057 (3)	0.061 (3)	0.038 (2)	−0.007 (3)	−0.001 (2)	0.007 (2)
N3	0.059 (2)	0.046 (2)	0.0407 (19)	−0.0101 (19)	−0.0035 (18)	0.0027 (17)
N4	0.055 (2)	0.034 (2)	0.0360 (17)	0.000 (2)	−0.0029 (17)	0.0021 (14)
C5	0.046 (2)	0.037 (2)	0.037 (2)	−0.003 (2)	−0.0002 (19)	0.009 (2)
C6	0.046 (2)	0.037 (3)	0.043 (2)	−0.007 (2)	0.004 (2)	0.003 (2)
N7	0.062 (2)	0.0325 (18)	0.0440 (18)	0.004 (2)	−0.0054 (19)	0.0006 (16)
C7A	0.057 (3)	0.036 (3)	0.040 (2)	0.001 (2)	−0.002 (2)	0.0116 (19)
C27	0.072 (3)	0.080 (4)	0.046 (3)	−0.021 (3)	−0.007 (2)	0.004 (3)
C21	0.070 (4)	0.064 (4)	0.041 (2)	−0.015 (5)	−0.016 (3)	−0.004 (4)
C22	0.088 (6)	0.061 (4)	0.066 (4)	−0.002 (4)	0.020 (4)	0.001 (3)
C23	0.095 (6)	0.074 (5)	0.066 (4)	−0.011 (4)	0.019 (4)	0.006 (4)
C24	0.057 (5)	0.103 (7)	0.056 (4)	−0.020 (5)	0.005 (3)	−0.017 (4)
Cl24	0.0763 (14)	0.165 (3)	0.0743 (12)	−0.0284 (17)	0.0220 (11)	−0.0164 (15)
C25	0.086 (6)	0.113 (7)	0.079 (5)	0.017 (8)	0.001 (4)	−0.012 (7)
C26	0.091 (7)	0.083 (5)	0.070 (4)	0.008 (5)	−0.010 (4)	0.008 (4)
C37	0.072 (3)	0.080 (4)	0.046 (3)	−0.021 (3)	−0.007 (2)	0.004 (3)
C31	0.070 (4)	0.064 (4)	0.041 (2)	−0.015 (5)	−0.016 (3)	−0.004 (4)
C32	0.088 (6)	0.061 (4)	0.066 (4)	−0.002 (4)	0.020 (4)	0.001 (3)
C33	0.095 (6)	0.074 (5)	0.066 (4)	−0.011 (4)	0.019 (4)	0.006 (4)
C34	0.057 (5)	0.103 (7)	0.056 (4)	−0.020 (5)	0.005 (3)	−0.017 (4)
Cl34	0.0763 (14)	0.165 (3)	0.0743 (12)	−0.0284 (17)	0.0220 (11)	−0.0164 (15)
C35	0.086 (6)	0.113 (7)	0.079 (5)	0.017 (8)	0.001 (4)	−0.012 (7)
C36	0.091 (7)	0.083 (5)	0.070 (4)	0.008 (5)	−0.010 (4)	0.008 (4)
C51	0.052 (3)	0.042 (3)	0.053 (3)	0.004 (2)	−0.006 (2)	0.001 (2)
N51	0.079 (3)	0.038 (2)	0.060 (2)	0.008 (3)	−0.017 (2)	−0.001 (2)
C52	0.061 (3)	0.044 (3)	0.050 (3)	0.008 (3)	−0.006 (2)	0.000 (2)
C53	0.051 (2)	0.040 (3)	0.041 (2)	0.007 (2)	−0.015 (2)	0.004 (2)
C53A	0.055 (3)	0.039 (2)	0.042 (2)	0.004 (2)	−0.010 (2)	0.005 (2)
C54	0.065 (3)	0.055 (3)	0.051 (3)	0.003 (3)	−0.006 (2)	0.004 (3)
C55	0.083 (4)	0.083 (4)	0.060 (3)	0.009 (4)	0.004 (3)	0.002 (3)
C56	0.076 (4)	0.087 (5)	0.076 (4)	0.001 (4)	0.005 (3)	0.027 (4)
C57	0.073 (4)	0.051 (3)	0.082 (4)	−0.005 (3)	−0.006 (3)	0.026 (3)
C57A	0.062 (3)	0.039 (3)	0.057 (3)	−0.001 (2)	−0.016 (2)	0.011 (2)
C61	0.042 (2)	0.042 (3)	0.038 (2)	−0.013 (2)	0.0017 (19)	0.0045 (19)
C62	0.053 (3)	0.063 (3)	0.047 (3)	−0.010 (3)	0.006 (2)	−0.004 (2)
C63	0.045 (3)	0.074 (3)	0.058 (3)	−0.004 (3)	−0.005 (3)	−0.003 (3)
C64	0.059 (3)	0.072 (3)	0.040 (2)	−0.019 (3)	−0.008 (2)	−0.003 (3)
Br64	0.0976 (4)	0.1173 (5)	0.0545 (3)	−0.0123 (4)	−0.0222 (3)	−0.0103 (3)
C65	0.064 (3)	0.072 (4)	0.042 (2)	−0.008 (3)	0.001 (2)	−0.012 (2)
C66	0.050 (3)	0.058 (3)	0.049 (2)	−0.002 (3)	0.001 (2)	0.004 (2)

### 6-(4-Bromophenyl)-2-(4-chlorobenzyl)-5-[(1H-indol-3-yl)methyl]imidazo[2,1-b][1,3,4]thiadiazole (III) Geometric parameters (Å, º)

**Table:**

S1—C7A	1.726 (4)	C35—C36	1.399 (14)
S1—C2	1.750 (5)	C35—H35	0.9300
C2—N3	1.283 (5)	C36—H36	0.9300
C2—C27	1.487 (6)	C51—C53	1.493 (6)
N3—N4	1.376 (5)	C51—H51A	0.9700
N4—C7A	1.363 (5)	C51—H51B	0.9700
N4—C5	1.381 (5)	N51—C52	1.362 (6)
C5—C6	1.376 (6)	N51—C57A	1.373 (6)
C5—C51	1.490 (6)	N51—H51	0.99 (5)
C6—N7	1.391 (5)	C52—C53	1.357 (6)
C6—C61	1.471 (6)	C52—H52	0.9300
N7—C7A	1.304 (5)	C53—C53A	1.426 (6)
C27—C21	1.511 (7)	C53A—C54	1.400 (6)
C27—H27A	0.9700	C53A—C57A	1.413 (7)
C27—H27B	0.9700	C54—C55	1.373 (7)
C21—C26	1.367 (11)	C54—H54	0.9300
C21—C22	1.373 (12)	C55—C56	1.393 (8)
C22—C23	1.387 (8)	C55—H55	0.9300
C22—H22	0.9300	C56—C57	1.367 (8)
C23—C24	1.369 (11)	C56—H56	0.9300
C23—H23	0.9300	C57—C57A	1.383 (7)
C24—C25	1.341 (11)	C57—H57	0.9300
C24—Cl24	1.746 (6)	C61—C62	1.374 (6)
C25—C26	1.402 (10)	C61—C66	1.378 (6)
C25—H25	0.9300	C62—C63	1.377 (6)
C26—H26	0.9300	C62—H62	0.9300
C31—C36	1.369 (14)	C63—C64	1.376 (7)
C31—C32	1.373 (17)	C63—H63	0.9300
C32—C33	1.395 (13)	C64—C65	1.358 (7)
C32—H32	0.9300	C64—Br64	1.906 (4)
C33—C34	1.368 (16)	C65—C66	1.385 (6)
C33—H33	0.9300	C65—H65	0.9300
C34—C35	1.340 (16)	C66—H66	0.9300
C34—Cl34	1.746 (12)		
			
C7A—S1—C2	88.0 (2)	C31—C36—C35	121.1 (16)
N3—C2—C27	122.1 (5)	C31—C36—H36	119.4
N3—C2—S1	117.1 (3)	C35—C36—H36	119.4
C27—C2—S1	120.8 (4)	C5—C51—C53	118.0 (3)
C2—N3—N4	108.1 (4)	C5—C51—H51A	107.8
C7A—N4—N3	117.9 (3)	C53—C51—H51A	107.8
C7A—N4—C5	108.2 (3)	C5—C51—H51B	107.8
N3—N4—C5	133.9 (3)	C53—C51—H51B	107.8
C6—C5—N4	103.2 (3)	H51A—C51—H51B	107.1
C6—C5—C51	134.1 (4)	C52—N51—C57A	108.2 (4)
N4—C5—C51	122.7 (4)	C52—N51—H51	117 (3)
C5—C6—N7	112.2 (4)	C57A—N51—H51	134 (3)
C5—C6—C61	127.2 (4)	C53—C52—N51	111.3 (4)
N7—C6—C61	120.5 (4)	C53—C52—H52	124.4
C7A—N7—C6	103.8 (3)	N51—C52—H52	124.4
N7—C7A—N4	112.6 (4)	C52—C53—C53A	106.0 (4)
N7—C7A—S1	138.6 (3)	C52—C53—C51	125.9 (4)
N4—C7A—S1	108.8 (3)	C53A—C53—C51	127.9 (4)
C2—C27—C21	112.2 (11)	C54—C53A—C57A	118.4 (4)
C2—C27—H27A	109.2	C54—C53A—C53	134.5 (4)
C21—C27—H27A	109.2	C57A—C53A—C53	107.1 (4)
C2—C27—H27B	109.2	C55—C54—C53A	118.9 (5)
C21—C27—H27B	109.2	C55—C54—H54	120.6
H27A—C27—H27B	107.9	C53A—C54—H54	120.6
C26—C21—C22	118.9 (6)	C54—C55—C56	121.8 (5)
C26—C21—C27	121.3 (8)	C54—C55—H55	119.1
C22—C21—C27	119.8 (8)	C56—C55—H55	119.1
C21—C22—C23	120.7 (6)	C57—C56—C55	120.5 (6)
C21—C22—H22	119.7	C57—C56—H56	119.7
C23—C22—H22	119.7	C55—C56—H56	119.7
C24—C23—C22	118.7 (6)	C56—C57—C57A	118.5 (5)
C24—C23—H23	120.6	C56—C57—H57	120.7
C22—C23—H23	120.6	C57A—C57—H57	120.7
C25—C24—C23	122.2 (6)	N51—C57A—C57	130.6 (5)
C25—C24—Cl24	119.9 (7)	N51—C57A—C53A	107.5 (4)
C23—C24—Cl24	118.0 (7)	C57—C57A—C53A	121.9 (5)
C24—C25—C26	118.5 (8)	C62—C61—C66	117.8 (4)
C24—C25—H25	120.7	C62—C61—C6	121.5 (4)
C26—C25—H25	120.7	C66—C61—C6	120.7 (4)
C21—C26—C25	120.9 (8)	C61—C62—C63	121.3 (5)
C21—C26—H26	119.5	C61—C62—H62	119.4
C25—C26—H26	119.5	C63—C62—H62	119.4
C36—C31—C32	118.0 (16)	C64—C63—C62	119.2 (5)
C31—C32—C33	120.9 (15)	C64—C63—H63	120.4
C31—C32—H32	119.5	C62—C63—H63	120.4
C33—C32—H32	119.5	C65—C64—C63	121.1 (4)
C34—C33—C32	118.8 (16)	C65—C64—Br64	120.0 (4)
C34—C33—H33	120.6	C63—C64—Br64	118.9 (4)
C32—C33—H33	120.6	C64—C65—C66	118.6 (5)
C35—C34—C33	121.2 (15)	C64—C65—H65	120.7
C35—C34—Cl34	118.7 (16)	C66—C65—H65	120.7
C33—C34—Cl34	119.7 (16)	C61—C66—C65	121.8 (4)
C34—C35—C36	119.5 (16)	C61—C66—H66	119.1
C34—C35—H35	120.3	C65—C66—H66	119.1
C36—C35—H35	120.3		
			
C7A—S1—C2—N3	0.9 (4)	C33—C34—C35—C36	2 (12)
C7A—S1—C2—C27	179.7 (4)	Cl34—C34—C35—C36	175 (5)
C27—C2—N3—N4	−179.2 (4)	C32—C31—C36—C35	3 (17)
S1—C2—N3—N4	−0.4 (5)	C34—C35—C36—C31	−1 (13)
C2—N3—N4—C7A	−0.5 (5)	C6—C5—C51—C53	−116.0 (5)
C2—N3—N4—C5	179.4 (4)	N4—C5—C51—C53	66.0 (5)
C7A—N4—C5—C6	−0.8 (4)	C57A—N51—C52—C53	−0.3 (5)
N3—N4—C5—C6	179.2 (4)	N51—C52—C53—C53A	−0.3 (5)
C7A—N4—C5—C51	177.7 (4)	N51—C52—C53—C51	−175.2 (4)
N3—N4—C5—C51	−2.3 (6)	C5—C51—C53—C52	−133.1 (4)
N4—C5—C6—N7	0.0 (4)	C5—C51—C53—C53A	53.1 (6)
C51—C5—C6—N7	−178.3 (4)	C52—C53—C53A—C54	−179.6 (5)
N4—C5—C6—C61	−176.0 (4)	C51—C53—C53A—C54	−4.8 (8)
C51—C5—C6—C61	5.7 (8)	C52—C53—C53A—C57A	0.8 (5)
C5—C6—N7—C7A	0.8 (5)	C51—C53—C53A—C57A	175.5 (4)
C61—C6—N7—C7A	177.2 (4)	C57A—C53A—C54—C55	1.3 (6)
C6—N7—C7A—N4	−1.4 (5)	C53—C53A—C54—C55	−178.3 (4)
C6—N7—C7A—S1	179.0 (4)	C53A—C54—C55—C56	0.2 (7)
N3—N4—C7A—N7	−178.6 (4)	C54—C55—C56—C57	−1.1 (8)
C5—N4—C7A—N7	1.5 (5)	C55—C56—C57—C57A	0.5 (8)
N3—N4—C7A—S1	1.2 (5)	C52—N51—C57A—C57	−177.9 (5)
C5—N4—C7A—S1	−178.8 (3)	C52—N51—C57A—C53A	0.8 (5)
C2—S1—C7A—N7	178.6 (5)	C56—C57—C57A—N51	179.4 (5)
C2—S1—C7A—N4	−1.1 (3)	C56—C57—C57A—C53A	0.9 (7)
N3—C2—C27—C21	89.1 (8)	C54—C53A—C57A—N51	179.3 (4)
S1—C2—C27—C21	−89.6 (7)	C53—C53A—C57A—N51	−0.9 (5)
C2—C27—C21—C26	−93.1 (19)	C54—C53A—C57A—C57	−1.9 (6)
C2—C27—C21—C22	87 (2)	C53—C53A—C57A—C57	177.8 (4)
C26—C21—C22—C23	−1 (3)	C5—C6—C61—C62	−41.3 (6)
C27—C21—C22—C23	179.7 (13)	N7—C6—C61—C62	142.9 (4)
C21—C22—C23—C24	−1.3 (16)	C5—C6—C61—C66	136.5 (5)
C22—C23—C24—C25	1.6 (11)	N7—C6—C61—C66	−39.2 (6)
C22—C23—C24—Cl24	−176.5 (5)	C66—C61—C62—C63	−4.0 (7)
C23—C24—C25—C26	0.0 (13)	C6—C61—C62—C63	173.9 (4)
Cl24—C24—C25—C26	178.0 (7)	C61—C62—C63—C64	1.6 (7)
C22—C21—C26—C25	2 (3)	C62—C63—C64—C65	2.1 (8)
C27—C21—C26—C25	−178.1 (14)	C62—C63—C64—Br64	−175.6 (4)
C24—C25—C26—C21	−1.9 (19)	C63—C64—C65—C66	−3.0 (8)
C36—C31—C32—C33	−7 (16)	Br64—C64—C65—C66	174.6 (4)
C31—C32—C33—C34	9 (10)	C62—C61—C66—C65	3.0 (7)
C32—C33—C34—C35	−6 (9)	C6—C61—C66—C65	−174.9 (4)
C32—C33—C34—Cl34	−178 (4)	C64—C65—C66—C61	0.5 (7)

### 6-(4-Bromophenyl)-2-(4-chlorobenzyl)-5-[(1H-indol-3-yl)methyl]imidazo[2,1-b][1,3,4]thiadiazole (III) Hydrogen-bond geometry (Å, º)

**Table:**

*D*—H···*A*	*D*—H	H···*A*	*D*···*A*	*D*—H···*A*
N51—H51···N7^i^	0.99 (5)	1.97 (5)	2.941 (5)	166 (4)
C51—H51*A*···*Cg*1^ii^	0.97	2.97	3.699 (5)	133
C62—H62···*Cg*2^ii^	0.93	2.91	3.757 (5)	152
C65—H65···*Cg*3^iii^	0.93	2.82	3.412 (7)	123
C65—H65···*Cg*4^iii^	0.93	2.91	3.60 (3)	131

Symmetry codes: (i) *x*, *y*+1, *z*; (ii) *x*−1/2, −*y*+3/2, −*z*+1; (iii) −*x*+3/2, −*y*+1, *z*−1/2.
